# Nest secondary plants and their associations with haemosporidian blood parasites in blue tit females

**DOI:** 10.1017/S0031182024000775

**Published:** 2024-09

**Authors:** Jorge García-Campa, Sonia González-Braojos, Judith Morales

**Affiliations:** 1 Department of Evolutionary Ecology, National Museum of Natural Sciences – Spanish National Research Council (CSIC). c/ José Gutiérrez Abascal 2, 28006 Madrid, Spain; 2 CIBIO-InBIO, Universidade do Porto, Campus de Vairão, Rua Padre Armando Quintas, 4485-661 Vairão, Portugal; 3 Instituto de Salud Carlos III. Carretera de Majadahonda – Pozuelo, Km. 2.200, 28220, Majadahonda, (Madrid), Spain

**Keywords:** aromatic plants, avian malaria, green plants, *Haemoproteus*, Haemosporidian blood parasites, *Leucocytozoon*, nesting behaviour, nest protection, *Plasmodium*, secondary plants

## Abstract

Avian nests often contain aromatic plant fragments, which has led to propose among others that they repel ectoparasites or vectors of blood parasites (‘nest protection hypothesis’). To date, the relationship between secondary plant provisioning and the parent's blood parasites remains unexplored. We investigated whether the presence of secondary plants in nests during different reproductive stages (before incubation, during incubation and nestling period) was associated with the presence of nest-dwelling ectoparasites and females’ blood-parasite infections in blue tits (*Cyanistes caeruleus*) during chick rearing. In this species, females are highly exposed to parasites, particularly at the beginning of the breeding season, since they build the nest and incubate alone. They also brood the nestlings while the male assists with provisioning. We found that females that provided fewer plants before incubation were more likely to be infected by *Plasmodium*. Specifically, Females that did not provide *Achillea* herbs before incubation were more likely to be infected by both *Plasmodium* and *Haemoproteus*, reinforcing the ‘nest protection’ hypothesis. Thus, secondary plants may create an early environment that masks the odour of hosts or repels vectors. Surprisingly, the presence of *Lavandula* during the nestling period was positively related to *Leucocytozoon* infection. Given its fastest development among haemosporidians, we speculate that *Lavandula* herbs are provided by females to reduce mother-to-offspring transmission or as a self-medication strategy. Finally, the number of plant fragments provided before incubation was negatively associated with the number of (*Protocalliphora azurea*) larvae, whereas there were no associations with the presence of mites (*Dermanyssus gallinoides*).

## Graphical abstract

**Figure:**
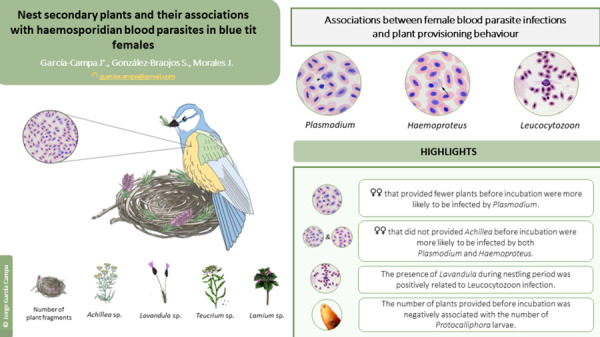

## Introduction

Nest building behaviour is widespread across avian species, being a critical stage that may influence the success or failure of reproduction (Mainwaring *et al*., 2023). Animals invest considerable time and effort in constructing nests that regulate temperature and humidity (Korb and Linsenmair, 1999), and provide shelter (Hansell, 2000) and protection for their offspring (Hansell, 2005). Nesting behaviour encompasses more than mere construction, as it usually involves the strategic incorporation of additional materials, such as hair, feathers or plants, which are not essential for the nest structure but may maximize reproductive success (Bush and Clayton, 2018). However, while the significance of nest structure and location has been extensively studied (Hansell, 2005; Mainwaring *et al*., 2014, 2023), the ecological role of additional nest materials, particularly those of plant origin, remains less explored.

Secondary plant fragments (hereafter ‘secondary plants’ for simplicity) that are deposited on the nest by some bird species represent a tiny percentage of the nest material (i.e. 4–13%; see Petit *et al*., 2002 in the blue tit *Cyanistes caeruleus*; Clark and Mason, 1985, in European starling *Sturnus vulgaris*; and Dykstra *et al*., 2009 in the red-shouldered hawk *Buteo lineatus*). Additionally, plant fragments are usually deposited on the nest cup or rim. Thus, they are in direct contact with the clutch, the incubating parent or the nestlings, and are clearly visible for the attending parents. Depending on the species, only males (e.g. European and spotless starling, *Sturnus unicolor*; Clark and Manson, 1985; Veiga *et al*., 2006), only females (e.g. blue tits, Petit *et al*., 2002), or both parents (e.g. broad-winged hawk, *Buteo platypterus*, Lyons *et al*., 1986; McCabe *et al*., 2019) provide secondary plants to their nests. For example, starling males build a nest mainly done with tree branches that they typically cover with a collection of green plants, hair and feathers (Gwinner, 1997; Veiga *et al*., 2006).

Historically, three non-mutually exclusive hypotheses have been proposed to explain why some bird species incorporate secondary plants to their nests. First, the ‘Courtship hypothesis’ holds that plant fragments function as a secondary sexual trait. For example, carrying of plants by starling males is synchronized with the females’ fertile period, presumably to stimulate them to breed (Gwinner, 1997; Gwinner *et al*., 2000; Brouwer and Komdeur, 2004; Polo *et al*., 2004; Veiga *et al*., 2006). In addition, carrying of secondary plants and nest size in blue tits has been suggested as sexual signals of the females to stimulate differential allocation in males (Gwinner, 1997; Tomás *et al*., 2013; Gwinner *et al*., 2000). The other two hypotheses are related to the fact that most secondary plants are aromatic and possess specific properties that enhance anti-parasite defences in birds. This might be particularly important in the nest environment of altricial birds due to the accumulation of nest materials, organic compounds and to a relatively high temperature, all of which favours the presence of nest parasites and blood-parasite vectors (Mainwaring *et al*., 2014; Scott-Baumann and Morgan, 2015).

Specifically, the ‘Nest protection hypothesis’ posits that plant metabolites, including volatile compounds, would reduce the load of nest parasites and blood-parasite vectors (Clark and Mason, 1985;, 1988; Clark, 1991; Dubiec *et al*., 2013; Scott-Baumann and Morgan, 2015). On the other hand, the ‘Drug hypothesis’ proposes that secondary plants directly promote offspring health and development *via* e.g. stimulation of the immune system (Gwinner *et al*., 2000), which allows individuals to better cope with parasites. Certainly, there is evidence that secondary plants constitute the first anti-parasite barrier, as their occurrence is negatively associated with the presence of blood-parasite vectors (Lafuma *et al*., 2001; Gwinner and Berger, 2006), nest-dwelling ectoparasites (Clark and Mason, 1988; Tomás *et al*., 2012) and bacteria (Mennerat *et al*., 2009). For example, the experimental addition of aromatic plants in blue tit nests, and particularly, of *Lavandula stoechas*, had a strong repellent effect against the mosquito *Culex pipiens*, and this and other plants such as *Achillea ligustica* had a masking effect on the chemical cues used by mosquitoes to find their hosts (Lafuma *et al*., 2001).

Given the severe consequences of nest parasites for nestling growth and survival (Hurtrez-Boussès *et al*., 1997; Simon *et al*., 2004; Thomas *et al*., 2007), various studies have focussed on exploring the effects of secondary plants on the nestlings. Indeed, in different bird species, the occurrence of secondary plants is associated with enhanced chick growth (Gwinner *et al*., 2000; Pires *et al*., 2020) and physiological condition (e.g. higher blood haemoglobin levels, Clark and Mason, 1988; Glądalski *et al*., 2020; higher haematocrit levels, Gwinner *et al*., 2000, Mennerat *et al*., 2009; longer telomere length, Soler *et al*., 2017), which supports both the ‘Nest protection hypothesis’ and the ‘Drug hypothesis’. However, not only the nestlings, but also the parents and, particularly, the mothers are highly exposed to nest parasites and blood-parasite vectors, given that they spend quite a considerable amount of time in the nest during construction, incubation and nestling provisioning. Accordingly, it has been observed that female great tits (*Parus major*) avoid sleeping in nests that have been experimentally infested with fleas (Christe *et al*., 1996). Furthermore, the exposure to blood-parasite vectors (i.e. biting midges) imposes high physiological costs on females (e.g. see Martínez-de la Puente *et al*., 2011 in the blue tit). However, although previous evidence suggest that blue tit females are selective in provisioning plants (Petit *et al*., 2002; Pires *et al*., 2012; Garrido-Bautista *et al*., 2023), the link between secondary plants and blood-parasite infection in adult birds remains unexplored.

The aim of this study was twofold. First, we investigated whether the blood-parasite infection (i.e. presence/absence of *Plasmodium*, *Haemoproteus,* and *Leucocytozoon*) of blue tit females was associated with the occurrence of particular plant genera in the nest during different stages of reproduction. Second, we explored the link between the different plant genera and the occurrence of two common nest-dwelling ectoparasites (i.e. blowfly pupae *Protocalliphora azurea* and mites *Dermanyssus gallinoides*). In the blue tit, females build the nest alone and show a marked preference for incorporating secondary plants in their nests. The provisioning of secondary plants involves a small percentage of the plant species available in the habitat (e.g. 5% in blue tits; Petit *et al*., 2002; Pires *et al*., 2012; Dubiec *et al*., 2013), suggesting that females are very selective and show a preference for particular plants. Most plant species gathered by females are aromatic (e.g. *Achillea* sp., *Lavandula* sp.; Pirmohammadi *et al*., 2016; Caputo *et al*., 2018) and characterised by a high content of secondary metabolites, including volatile chemicals known for their anti-parasitic and anti-pathogenic properties (Hart, 1997; see also Ruiz-Castellano *et al*., 2016 in spotless starling).

We predicted that females that provide more plant fragments to their nests are less likely to show blood-parasite infection, given that secondary plants can repel blood-parasite vectors (‘Nest-protection hypothesis’) and can also directly promote immune responsiveness and condition (‘Drug hypothesis’ as applied to adult birds). In particular, we expected that blood-parasite prevalence should be more strongly associated with plant provisioning behaviour at early stages of reproduction rather than during the nestling period, given that females spend more time in the nest (and are thus more exposed to blood-parasite vectors) during egg laying and incubation. Similarly, we expected that the provisioning of secondary plants would reduce the development of nest-dwelling ectoparasites.

## Material and methods

### Study area and model species

The study was carried out in the locality of Miraflores de la Sierra, Madrid, central Spain (40°48′N, 03°47′W) throughout the breeding season of 2017. We studied a blue tit population breeding in nest-boxes in a deciduous forest at 1200 m a.s.l., mainly dominated by Pyrenean oak (*Quercus pyrenaica*). The blue tit is a socially monogamous passerine with intense bi-parental care. However, nest building is done by the female. The nests are built with moss and are lined with soft material such as hair and feathers. Additionally, blue tit females in the study population usually include pieces of other green plants, which they mostly incorporate when the nest is almost finished, and during egg laying and incubation of the eggs (Bańbura *et al*., 1995; Mainwaring, 2017). However, unlike other species like starlings, the provisioning of secondary plants continues during the whole breeding process (i.e. from the end of nest construction until fledging; Mennerat *et al*., 2009). The blue tit has one of the largest clutch sizes and the largest variation in clutch size among all European passerines (in our study population; mean ± s.d.: 9.6 ± 1.8 eggs; n = 464 clutches; range 4–15). The incubation stage ranges between 14 and 16 days (von Haartmann 1969; Salvador, 2016).

### General procedures

At the beginning of the breeding season, we started visiting nest-boxes to record the onset of nest construction. Once nest construction was at an advanced stage, we inspected the nests every other day when possible (mean ± s.d. = 11.27 ± 2.91 inspections; *n* = 110 nests) in order to study the occurrence of secondary plants (see *Identification and quantification of secondary plants*). We also recorded the laying date, the onset of incubation and the hatching date (=day 0).

At days 9–12, we trapped blue tit females in their nest-boxes, weighted them using a spring balance (accuracy 0.01 g), and collected a blood sample from the brachial vein with a sterilized needle and a heparinized capillary. Then, we made a blood smear for blood parasite identification (see *Identification of blood parasites*). Blood smears were air-dried and fixed on the same day using absolute methanol for 1 min. After such fixation, they were stained for 45 min with Giemsa diluted 1:10 in buffer, pH 7.2 (Schall, 1986).

After fledging (i.e. day 20 approx.), all the nest material was collected in a sealed labelled plastic bag and kept at 4°C. Once in the laboratory, each nest was crumbled by hand on white paper to assess the presence/absence of two main nest dwelling parasites in blue tit nests: blowfly pupae of the dipteran *P. azurea*, which were also counted, and mites *D. gallinoides*. Additionally, we searched for all the dry plant fragments that remained in the nest after reproduction and weighed them with a high-precision balance (accuracy 0.001 g).

During the breeding season, we performed a parallel study, in which we placed feeders inside nest-boxes to supplement females with carotenoids (mainly lutein mixed with fat) prior and during egg laying (for details, see García-Campa *et al*., 2020 and Supplementary Material). The experimental treatment (lutein-supplemented and control) was initially controlled for in all statistical analyses, but since it was never significant (all *P* > 0.10), we excluded it from the final models (see Table S2).

### Identification and quantification of secondary plants

We recorded the provisioning of secondary plants at three stages of reproduction: before incubation (i.e. from the end of nest building to the onset of incubation; mean ± s.d. = 7.16 ± 2.53 visits), during incubation (i.e. from the onset of incubation to the hatching day; 2.61 ± 1.07 visits), and during the nestling period (i.e. from the hatching day to fledging; 1.51 ± 0.70 visits). At each visit, the different pieces of secondary plants found in the nest cup were placed on a white sheet of white paper, and were photographed together with a reference of 1 cm (1 picture per nest) for later count and identification in the laboratory. Plant fragments were placed back on the nest immediately after photographing them. In the field, it was not possible to weigh them accurately with portable balances, since each fragment usually weights less than 0.05 g. Thus, as mentioned above, we weighted them dry after nest collection, and once in the lab with a high precision balance (accuracy 0.001 g). We then used the photographs to count the total number of fragments found in each visit, regardless of plan genera. In addition, when possible, each fragment was assigned to a plant genus using a dichotomous key (General key to the Iberian flora – http://www.floraiberica.org/). However, identification of all fragments was not possible in some nests (26.9% before incubation, 20.9% during incubation, and 63.3% during the nestling period) and thus we decided to use the presence/absence of each plant genera instead of the number of fragments per genera.

To summarize, for each nest, we calculated the following three variables:

- Mean number of fragments (i.e. total number of fragments, regardless of the plant genera, found in each visit divided by the number of visits made), separately for the three periods (before incubation, incubation and nestling stage).
- Presence/absence of particular plant genera, separately for the three periods (before incubation, incubation and nestling stage).
- ‘Final dry mass’ (i.e. total dry mass of all the plant fragments extracted from the nest material after fledging).

### Identification of blood parasites

A half of each blood smear was scanned under 200× magnification in search of the extra-erythrocytic parasite *Trypanosoma* and the large intracellular *Leucocytozoon* However, only 3% of the females were infected by *Trypanosoma* and thus we did not analyse infection status by this parasite. In the other half of the blood smear, we scanned 50 fields under 1000× magnification using oil immersion, in search of intracellular parasites such as *Plasmodium* and *Haemoproteus* spp (see Merino *et al*., 1997; Morales *et al*., 2006).

### Sample size and statistical analyses

We observed the provisioning of secondary plants in 110 blue tit nests. However, we could identify blood parasites in 67 females, given that some nests failed after laying or because females could not be trapped or blood sampled. The presence/absence of nest-dwelling ectoparasites (i.e. *Protocalliphora* and *Dermanyssus*) was assessed in 65 nests, since two of the nests failed in the second week after hatching and were thus not comparable with the rest.

We used R 4.1.0 (R Core Team 2020) for statistical analyses. We investigated whether the presence/absence of each blood parasite (i.e. *Plasmodium*, *Haemoproteus* and *Leucocytozoon*) and of each nest-dwelling ectoparasite (i.e. the presence/absence and the abundance of *Protocalliphora* and the presence/absence of *Dermanyssus*), was related with the (i) mean number of secondary plant fragments, (ii) presence/absence of particular plant genera, and (iii) final dry mass of secondary plants, during the three different stages (i.e. before incubation, incubation and nestling). For this, we performed separate generalized linear models for the presence/absence of each parasite, which was included as the dependent variable, using binomial distribution and logit link function. We ran different models for each variable describing the plant provisioning behaviour (see above i, ii and iii), which was included as a covariate. Additionally, for the number of blowflies, we run generalized linear models with negative binomial distribution. In all models, we additionally included the female's body mass (g) and the laying date (Julian calendar) as covariates to control for female condition and possible effects of reproductive phenology.

## Results

### Provisioning behaviour of secondary plants

During the whole reproductive period, we found at least one plant fragment in 103 out of the 110 nests that we followed (95.4% of nests). Separately, for each stage, we found at least one plant fragment in 58.5% of nests before incubation, 57.7% during incubation, and 88.1% during the nestling period. Similarly, for the subset of nests with female's blood parasite information, we found at least one plant fragment in 65 out of 67 nests (97.0% of nests). For each stage, in this subset of nests, we found at least one plant fragment in 58.2% of nests before incubation, 61.2% during incubation, and 92.4% during the nestling period.

We identified seven plant genera: *Lavandula* sp. (at least once in 67.2% nests), *Anthriscus* sp. (49.3%), *Thymus* sp. (35.8%), *Achillea* sp. (31.3%), *Teucrium* sp. (26.9%), *Lamium* sp. (23.9%) and *Clinopodium* sp. (13.4%). The presence of *Lavandula* sp. clearly increased during reproduction, being more than double throughout the nestling period than before incubation, while the ocurrence of *Achillea* sp. and *Teucrium* sp. remained more or less stable (Table 1). *Anthriscus* sp. was reduced during incubation, while *Lamium* sp. decreased throughout reproduction. *Thymus* sp. was only abundant during the nestling period and thus we did not analyse its presence at prior stages (Table 1). Finally, *Clinopodium* sp. was very scarce and we did not include it in further analyses (Table 1).

**Table 1.** Provisioning behaviour of secondary plant species

Species	Total (%)	Before incubation (%)	Incubation (%)	Nestling period (%)
*Lavandula* sp.	67.2	19.4	26.9	60.6
			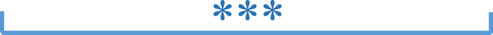	
		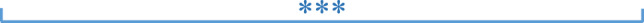		
*Anthriscus* sp.	49.3	29.9	14.9	28.8
		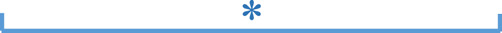		
*Thymus* sp.	35.8	4.5	6.2	31.8
			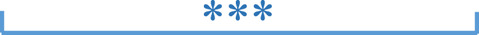	
		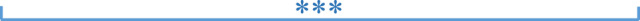		
*Achillea* sp.	31.3	14.9	17.9	12.1
*Teucrium* sp.	26.9	10.5	9.0	9.1
*Lamium* sp.	23.9	14.9	10.5	4.5
		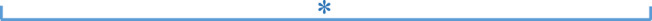		
*Clinopodium* sp.	13.4	2.9	1.5	9.1

Percentages of appearance of the 7 plan genera (*Lavandula* sp., *Anthriscus* sp., *Thymus* sp., *Achillea* sp., *Teucrium* sp., *Lamium* sp., and *Clinopodium* sp.) found in blue tit nests during the different periods: before the incubation, during the incubation, nestling period and total. Significant associations between periods are marked with an asterisk (* for *P* < 0.05 and *** for *P* < 0.001. *P* values were obtained from chi square tests). For detailed statistics, see Supplementary Table S1.

### Association between blood parasites and secondary plants

**Figure 1.**
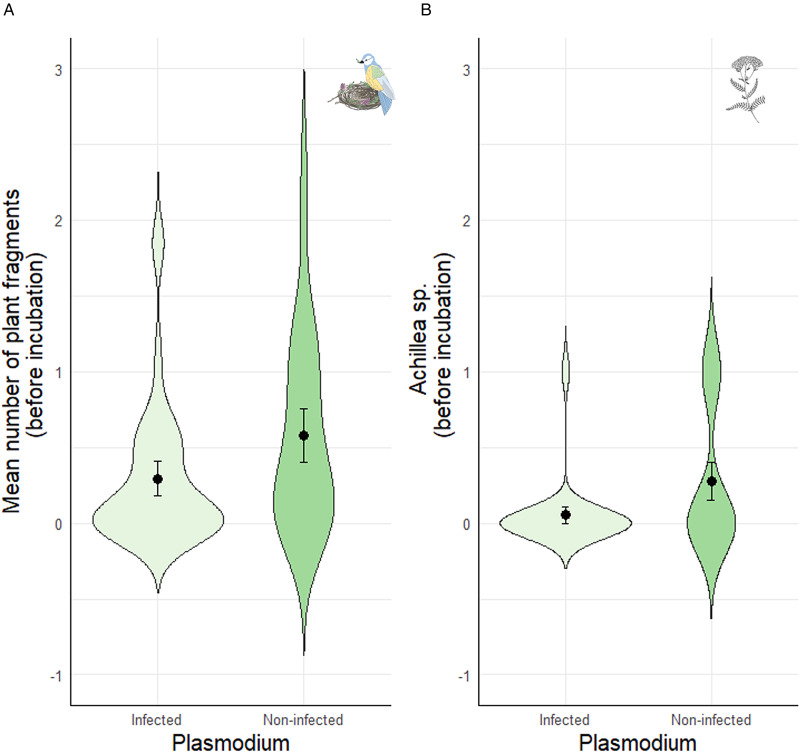
Relationship between the prevalence of *Plasmodium* infection in blue tit females with (a) the mean number of plant fragments before incubation, and (b) the presence of *Achillea* sp. fragments before incubation. Values are means ± SE.

**Figure 2.**
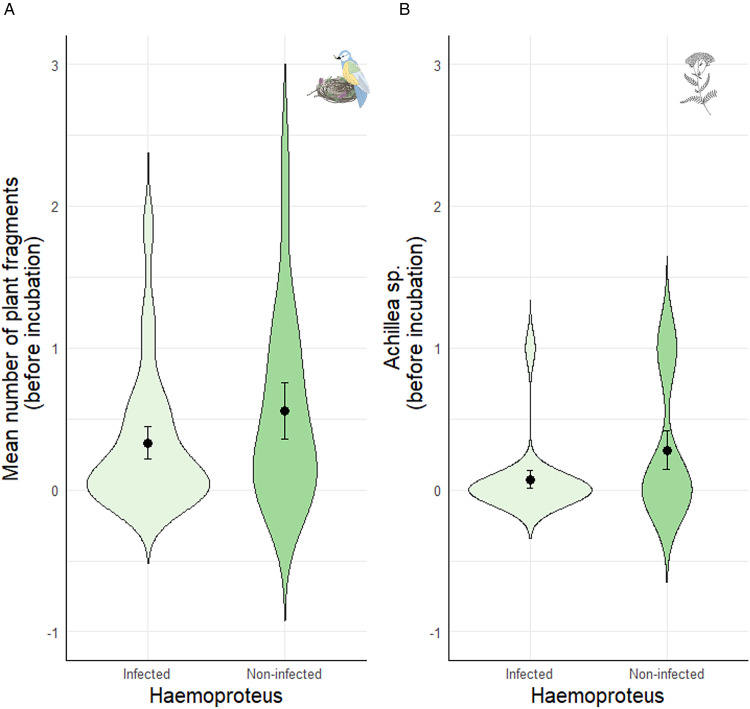
Relationship between the prevalence of *Haemoproteus* infection in blue tit females with (a) the mean number of plant fragments before incubation, and (b) the presence of *Achillea* sp. herbs before incubation. Values are means ± SE.

Fifty out of 67 females (74.63%) were infected by at least one blood parasite (38 females by *Plasmodium*, 42 by *Haemoproteus* and 15 by *Leucocytozoon*). Regardless of the plant genera, the presence/absence of *Plasmodium* was associated with the mean number of plant fragments before incubation, but not at other reproductive stages (Table S2). Specifically, females that provided on average more plant fragments before incubation were less likely to be infected by *Plasmodium* later on (Table S2; Figure 1a). The presence/absence of *Haemoproteus* or *Leucocytozoon* was not associated with the mean number of fragments at any reproductive stage (Table S2), although females that provided more plant fragments before incubation were marginally (and non-significantly) less likely to be infected by *Haemoproteus* (Table S2; Figure 2a). Female body mass or laying date were not associated with the presence/absence of any of the blood parasites (all *P* > 0.25 for female mass and all *P* > 0.07 for laying date; Table S2; see also Table S3).

**Figure 3.**
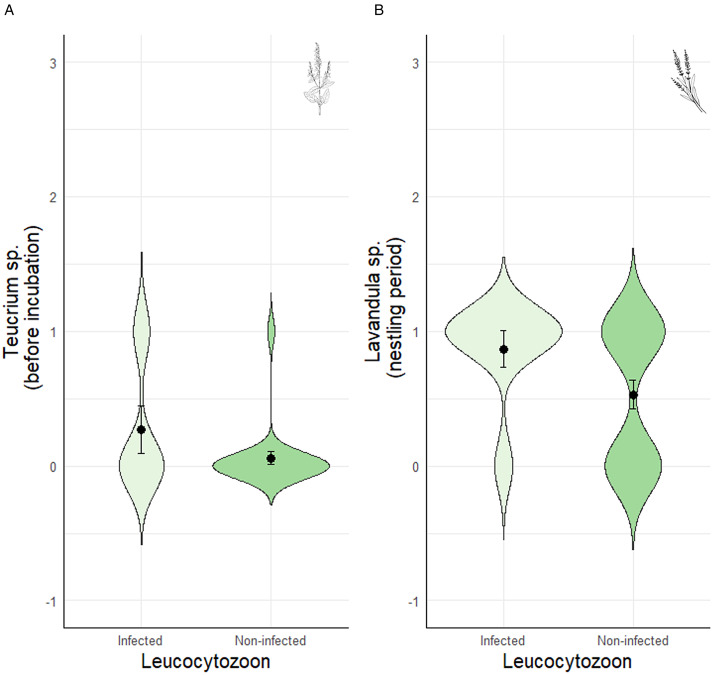
Relationship between the prevalence of *Leucocytozoon* infection in blue tit females with the presence of (a) *Teucrium* sp. herbs before incubation, and (b) *Lavandula* sp. herbs during the nestling period. Values are means ± SE.

When analysing the presence/absence of the different plant genera and their association with blood parasites, we found that females were less likely to be infected by *Plasmodium* when they had included *Achillea* sp. in the nest before incubation (Table 2a; Figure 1b). This association remained significant throughout incubation (Table 2b), but not in the nestling period (Table 2c). Infection by *Plasmodium* was not associated with the occurrence of other plant genera (Table 2). Similarly, females were less likely to be infected by *Haemoproteus* when they had included *Achillea* sp. before incubation (Table 2a; Figure 2b). Furthermore, females that provided *Lavandula* sp. fragments during incubation were also less likely to be infected by *Haemoproteus*, although in this case the relationship was marginally non-significant (Table 2b). On the contrary, females were more likely to be infected by *Leucocytozoon* when they had included *Teucrium* sp. fragments in the nests before incubation (Table 2a; Figure 3a), and *Lavandula* sp. fragments during the nestling period (Table 2c; Figure 3b). The rest of associations was not significant (Table 2).

**Table 2.** Generalized linear models (GLMs) with binomial error distribution showing the relationships between the presence/absence of particular plant genera during 3 reproductive stages and (i) the females’ haemosporidian infection status (i.e. *Plasmodium*, *Haemoproteus* and *Leucocytozoon*) and (ii) nest-dwelling ectoparasites (i.e. *Protocalliphora* and *Dermanyssus*)

	Plasmodium	Haemoproteus	Leucocytozoon	Protocalliphora	Dermanyssus
a) Before incubation					
*Achillea* sp.	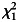 = 5.73 *coef.* = −1.82 ± 0.85 ***P* = 0.031**	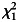 = 4.79 *coef*. = −1.58 ± 0.76 ***P* = 0.037**	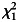 = 1.23 *coef*. = −1.11 ± 1.12 *P* = 0.32	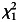 = 2.61 *coef*. = −1.20 ± 0.77 *P* *=* 0.12	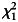 = 1.22 *coef*. = −1.10 ± 1.11 *P* *=* 0.32
*Lavandula* sp.	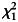 = 0.76 *coef*. = −0.56 ± 0.64 *P* = 0.38	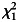 = 0.01 *coef*. = −0.07 ± 0.65 *P* = 0.92	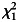 = 0.21 *coef*. = 0.33 ± 0.72 *P* = 0.64	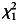 = 0.07 *coef*. = 0.17 ± 0.65 *P* = 0.79	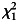 = 1.19 *coef*. = −0.86 ± 0.84 *P* = 0.31
*Anthriscus* sp.	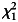 = 0.20 *coef*. = −0.25 ± 0.56 *P* = 0.66	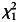 = 0.28 *coef*. = −0.30 ± 0.57 *P* = 0.60	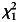 = 0.67 *coef*. = 0.52 ± 0.64 *P* = 0.41	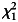 = 0.27 *coef*. = 0.30 ± 0.58 *P* = 0.61	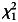 = 0.49 *coef*. = −0.46 ± 0.67 *P* = 0.49
*Teucrium* sp.	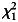 = 2.43 *coef*. = −1.32 ± 0.89 *P* = 0.14	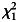 = 1.21 *coef*. = −0.90 ± 0.82 *P* = 0.28	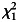 = 4.04 *coef*. = 1.75 ± 0.87 *P* = **0.045**	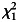 = 2.78 *coef*. = −1.41 ± 0.89 *P* = 0.12	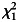 = 1.00 *coef*. = 0.85 ± 0.83 *P* = 0.31
*Lamium* sp.	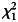 = 0.22 *coef*. = −0.34 ± 0.72 *P* = 0.64	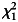 = 0.23 *coef*. = 0.36 ± 0.77 *P* = 0.64	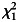 = 2.76 *coef*. = 1.26 ± 0.75 *P* = 0.09	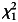 = 4.36 *coef*. = −1.57 ± 0.80 ***P* = 0.048**	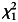 = 1.31 *coef*. = 0.89 ± 0.76 *P* = 0.25
b) Incubation					
*Achillea* sp.	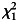 = 4.60 *coef.* = −1.51 ± 0.75 ***P* = 0.044**	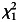 = 1.45 *coef*. = −0.81 ± 0.67 *P* = 0.23	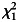 = 0.13 *coef*. = −0.30 ± 0.86 *P* = 0.73	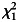 = 0.20 *coef*. = 0.30 ± 0.69 *P* = 0.66	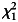 = 0.61 *coef*. = −0.63 ± 0.84 *P* = 0.46
*Lavandula* sp.	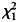 = 0.07 *coef*. = 0.16 ± 0.60 *P* = 0.79	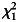 = 3.88 *coef*. = −1.19 ± 0.62 *P* = 0.054	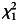 = 0.36 *coef*. = 0.41 ± 0.68 *P* = 0.55	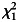 = 0.65 *coef*. = 0.49 ± 0.62 *P* = 0.43	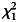 = 0.55 *coef*. = −0.51 ± 0.70 *P* = 0.45
*Anthriscus* sp.	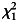 = 0.07 *coef*. = 0.19 ± 0.72 *P* = 0.79	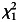 = 2.02 *coef*. = 1.12 ± 0.85 *P* = 0.19	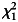 = 0.003 *coef*. = 0.05 ± 0.88 *P* = 0.96	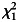 = 0.79 *coef*. = −0.66 ± 0.75 *P* = 0.38	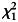 = 0.74 *coef*. = 0.26 ± 0.79 *P* = 0.74
*Teucrium* sp.	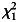 = 0.01 *coef*. = 0.11 ± 0.97 *P* = 0.91	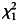 = 0.03 *coef*. = −0.18 ± 0.97 *P* = 0.85	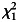 = 2.61 *coef*. = 1.61 ± 1.00 *P* = 0.11	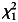 = 0.001 *coef*. = 0.04 ± 0.97 *P* = 0.97	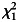 = 0.52 *coef*. = 0.73 ± 0.98 *P* = 0.46
*Lamium* sp.	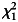 = 0.01 *coef*. = −0.07 ± 0.83 *P* = 0.93	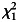 = 0.15 *coef*. = −0.33 ± 0.83 *P* = 0.70	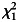 = 0.70 *coef*. = −0.90 ± 1.16 *P* = 0.44	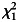 = 0.49 *coef*. = −0.61 ± 0.90 *P* = 0.50	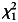 = 0.97 *coef*. = 0.86 ± 0.85 *P* = 0.32
c) Nestling					
*Achillea* sp.	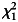 = 1.42 *coef*. = −0.93 ± 0.80 *P* = 0.24	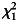 = 0.01 *coef*. = 0.06 ± 0.78 *P* = 0.94	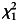 = 4.73 *coef*. = −17.59 ± 2252.54 *P* = 0.99	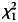 = 0.01 *coef*. = 0.06 ± 0.82 *P* = 0.94	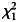 = 4.63 *coef*. = −16.72 ± 1493.68 *P* = 0.99
*Lavandula* sp.	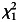 = 0.95 *coef*. = 0.51 ± 0.53 *P* = 0.33	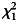 = 0.19 *coef*. = −0.23 ± 0.54 *P* = 0.66	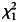 = 6.75 *coef*. = 1.91 ± 0.85 ***P* = 0.025**	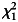 = 0.07 *coef*. = 0.14 ± 0.53 *P* = 0.79	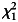 = 2.05 *coef*. = 0.90 ± 0.65 *P* = 0.17
*Anthriscus* sp.	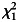 = 0.40 *coef*. = 0.36 ± 0.57 *P* = 0.53	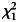 = 0.13 *coef*. = −0.21 ± 0.56 *P* = 0.71	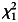 = 1.15 *coef*. = 0.69 ± 0.64 *P* = 0.28	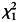 = 0.33 *coef*. = −0.32 ± 0.56 *P* = 0.57	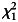 = 0.02 *coef*. = −0.08 ± 0.63 *P* = 0.89
*Teucrium* sp.	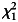 = 0.17 *coef*. = −0.35 ± 0.87 *P* = 0.69	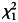 = 0.38 *coef*. = −0.54 ± 0.87 *P* = 0.54	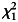 = 0.69 *coef*. = −0.45 ± 1.15 *P* = 0.70	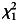 = 0.00 *coef*. = 0.40 ± 0.91 *P* = 0.66	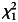 = 0.11 *coef*. = 0.31 ± 0.92 *P* = 0.73
*Lamium* sp.	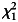 = 3.70 *coef*. = 16.51 ± 1335.99 *P* = 0.99	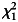 = 2.82 *coef*. = 16.15 ± 1377.13 *P* = 0.99	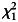 = 1.42 *coef*. = 1.52 ± 1.32 *P* = 0.25	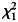 = 3.44 *coef*. = 16.42 ± 1365.41 *P* = 0.99	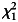 = 1.77 *coef*. = −15.57 ± 1377.06 *P* = 0.99
*Thymus* sp.	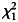 = 0.27 *coef*. = −0.28 ± 0.54 *P* = 0.61	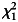 = 2.33 *coef*. = −0.84 ± 0.55 *P* = 0.13	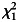 = 1.17 *coef*. = −0.76 ± 0.73 *P* = 0.30	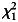 = 0.37 *coef*. = −0.33 ± 0.55 *P* = 0.55	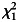 = 1.74 *coef*. = 0.78 ± 0.59 *P* = 0.19

*Thymus* sp. is only included for the nestling period due to the low sample size before hatching. Significant effects (*P* < 0.05) are marked in bold. Whole models including female body mass and laying date are shown in Table S3 and S4.

### Association between ectoparasites and secondary plants

We found *Protocalliphora* pupae in 37 out of the 65 nests (56.9% of nests with female's parasite status). Interestingly, the abundance of *Protocalliphora* pupae was associated with the mean number of plant fragments before incubation (Table S2), but not at other reproductive stages (Table S2). Mites were present in 17 out of the 65 nests (26.1%), and their occurrence was not associated with the mean number of plant fragments provided (Table S2).

**Figure 4.**
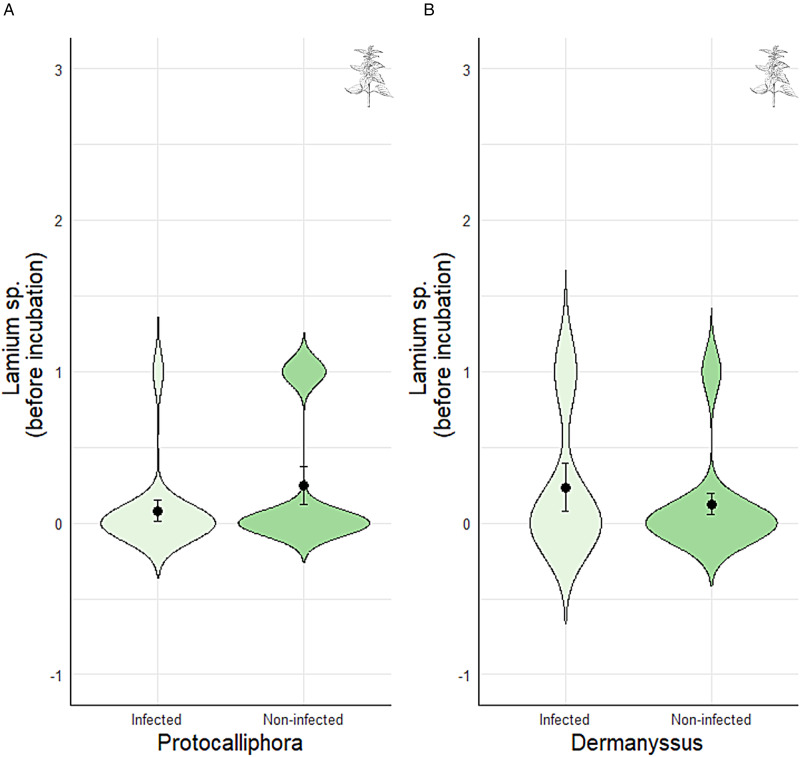
Relationship between the presence of (a) *Procalliphora azurea* pupae and (b) *Dermanyssus gallinoides* with the presence of *Lamium* sp. fragments before the incubation period. Values are means ± SE.

When we analysed the occurrence of particular plant genera, nests in which females provided *Lamium* sp. herbs before incubation were less likely to be infected by *Protocalliphora* pupae (Table 2a; Figure 4a). However, the presence of *Dermanyssus* was not related with the presence/absence of *Lamium* sp. (Table 2; Figure 4b) or with any other plan genus (Table 2).

## Discussion

In this study, we investigated whether the provisioning behaviour of secondary plants in the blue tit, a bird species in which females alone build the nest, is associated with the female's blood-parasite infection and with the presence of ectoparasites in the nest. Historically, studies on the role of secondary plants in avian nests have focussed mainly on their antimicrobial and insect-repellent properties (Lafuma *et al*., 2001; Tomás *et al*., 2012; Ruiz-Castellano *et al*., 2016), and thus on their potential beneficial effect on the nestlings (Gwinner *et al*., 2000; Soler *et al*., 2017; Gwinner *et al.*, 2018). However, no study to date has investigated the association between secondary plants and the female's blood-parasite status.

As hypothesized, we found certain evidence for the above association, although it varied among the three blood parasites studied and across the different stages of reproduction. On the one hand, the prevalence of *Haemoproteus* and *Leucocytozoon* was apparently not related to the mean number of plant fragments provided. On the other hand, regardless of the plant genera, females that were infected with *Plasmodium* at the end of the reproductive season had significantly provided less plant fragments to the nest before incubation than non-infected females. However, we did not find differences between these two groups of females after the onset of incubation. Following the ‘Nest protection hypothesis’, if secondary plants exert a repellent role against potential vectors of blood parasites, the provisioning of more plant fragments before incubation could have prolonged the time period during which the plant repellent properties were active and, thus, would have reduced the female's chances of being infected. Indeed, blue tit females spend more time in the nest during the onset of the breeding season and are therefore potentially more vulnerable to blood-parasite vectors at early stages. We expected that the amount of plant fragments would also be relevant during incubation, but our results suggest that the early nest environment (i.e. during nest construction and egg laying) is determinant for the female's parasitic status. Moreover, the estimated time of detection of *Plasmodium* in birds’ blood is at least 5 days from infection (i.e. time to maturation of the first generation of metacryptozoites in *P. relictum*; Huijben *et al*., 2007), making it less likely that the prevalence of *Plasmodium* is affected by plant provisioning during the nestling period. Analysing the female's parasite status at the beginning of the study would have allowed testing this possibility. Unfortunately, females are more prone to desert their nest when blood sampled before incubation and, thus, we only captured them once.

Additionally, following the ‘Drug hypothesis’, females would have benefited from the immune-stimulant properties of secondary plants, when dealing with *Plasmodium.* Although we expected that the amount of plant fragments would be associated with the prevalence of the 3 haemoparasites, it is possible that the immuno-stimulant properties of secondary plants have a more prominent role against infections by *Plasmodium*, or that their repellent role is more effective against their vectors (i.e. species of Culicidae family as *Aedes*, *Anopheles* and *Culex*; Valkiunas, 2004, LaPointe *et al*., 2005, Martínez-de la Puente *et al*., 2009).

Nonetheless, when analysing the presence of particular plant genera, we found various significant associations with the 3 avian haemosporidian parasites (see below). In total, we identified 7 plant genera provided by blue tit females, all of them defined as aromatic herbs, and their occurrence being similar than that observed in other studies in the blue tit (Petit *et al*., 2002). Interestingly, the presence of *Achillea* sp. herbs in the nests was negatively related to the female's blood-parasite infection, at least for *Plasmodium* and *Haemoproteus*. Females that had not provided *Achillea* sp. herbs before incubation were more likely to be infected by either *Plasmodium* or *Haemoproteus* than females with *Achillea* sp. fragments in their nest. Moreover, the relationship with *Plasmodium* infection remained significant during incubation, although it was relatively weaker. *Achillea* sp. are broadly known for their wide range of secondary metabolites (e.g. more than 50 volatile compounds identified from the essential oil of *Achillea* sp.; Al-Snafi, 2013), which have been traditionally used for their insect repellent properties and their antimicrobial, antioxidant and cytotoxic effects (Ahmadi, 2011; Al-Snafi, 2013; Pirmohammadi *et al*., 2016). The abundance of *Achillea* sp. in the study area is lower at the beginning of the reproductive season and peaks during June-July GBIF.org, 2023; https://www.gbif.org). Yet, its occurrence in blue tit nests remained stable and even decreased slightly during the nestling period (i.e. *Achillea* sp. before incubation: 14.9%; incubation: 17.9%; nestling period: 12.1%). This implies that gathering *Achillea* herbs during the early stages of the breeding season might be more difficult for breeding females, but might create a nest environment that helps them to cope with blood-parasite infections.

Surprisingly, females that had provided *Lavandula* sp. herbs during the nestling period were more likely to be infected by *Leucocytozoon*. Additionally, the presence of *Teucrium* before the incubation was also positively related with *Leucocytozoon* infection. However, the latter results should be taken with caution given the low number of females providing *Teucrium* to their nest. Considering that *Leucocytozoon* has the fastest meront-to-gametocyte development among bird haemosporidians (i.e. 10–12 days; Valkiunas and Iezhova, 2018), we may speculate that females infected by *Leucocytozoon* provided *Lavandula* sp. to their nest in order to repel vectors and thus reduce mother-to-offspring transmission (Ashford *et al*., 1990; Chakarov *et al*., 2015). Indeed, prior research has shown vector-mediated parent-to-offspring transmission in common buzzards (*Buteo buteo*, Chakarov *et al*., 2015). This could explain why females doubled the provisioning of *Lavandula* sp. in the nest during the nestling period. The main constituent of *Lavandula* sp. essential oil is the linalool, a terpene with anti-protist properties, including antimalarial effects (Silva *et al*., 2015; Caputo *et al*., 2018; Gabriel *et al*., 2018), and, indeed, it inhibits the development of parasitic protists such as *Plasmodium*, *Haemoproteus* and *Leucocytozoon* (Goulart *et al*., 2004; Guggisberg *et al*., 2014). Furthermore, *Thymus* sp. is also characterized by its linalool and thymol components (Rasooli and Mirmostafa, 2003; Rota *et al*., 2008; Boaventura *et al*., 2022), and was notably more abundant during the nestling period too. An additional possibility is that infected females provided aromatic herbs as a self-medication strategy against *Leucocytozoon* infection (Lozano, 1998; De Roode *et al*., 2013; Bravo *et al*., 2014).

An alternative explanation for our results is that females infected by *Plasmodium* and *Haemoproteus* were in worse condition and, therefore, they were not able to seek specific plants or to provide as many plant fragments to their nest as uninfected females. This would support a trade-off between condition or immune status and the allocation of plant resources to the nest. Indeed, provisioning behaviour of secondary nest materials is highly demanding in terms of energy and time (Moreno *et al*., 1994). Nonetheless, female body mass, as a proxy of female condition, was not associated with blood-parasite status, which does not support the possible trade-off (Tables S2 and S3). Moreover, the higher provisioning of *Lavandula* and *Teucrium* by *Leucocytozoon*-infected females, perhaps as a self-medication strategy or as a way to reduce nestling infection, does not support this explanation either. Although we cannot totally discard the existence of a trade-off between condition and plant provisioning behaviour, we consider the repellent role of secondary plants against blood-parasite vectors as a stronger possibility. Assessing the relationship between immune responsiveness and aromatic plants would have helped to tease apart these possibilities. Further studies measuring immune responsiveness (e.g. by inducing immune activation to a novel antigen; e.g. as in Morales *et al*., 2004, 2006; Tomás *et al*., 2007) could shed light in in this question.

According to the ‘Nest protection hypothesis’, we predicted that secondary plant provisioning would prevent the development of nest-dwelling ectoparasites (i.e. blowfly pupae *P. azurea* and mites *D. gallinoides*). However, contrary to previous studies (reviewed in Scott-Baumann and Morgan, 2015; Yang *et al*., 2020; Garrido-Bautista *et al*., 2023), the occurrence of mites in the nest was not associated with the mean number of fragments provided (but see Scott-Baumann *et al*., 2022), nor with the occurrence of any secondary plant genus. It is possible, however, that the presence of plant fragments reduces the level of infestation by ectoparasites (i.e. see Martínez-de la Puente *et al*., 2011; Tomás *et al*., 2012), which we did not measure. If we had explored mite abundance/intensity rather than mite prevalence, perhaps we would have found a significant association. Interestingly, however, we did find a significant association with the number of blowflies, which was higher in nests with less plant fragments before incubation. These results partly support our initial prediction and are consistent with the ‘Nest protection hypothesis’. Moreover, when exploring particular plant genera and the presence/absence of blowflies, we found that nests containing *Lamium* sp. fragments before incubation were less likely to be infested by *Protocalliphora*, which may suggest that *Lamium* herbs create an adverse nest environment for the development of *Protocalliphora* larval stages (e.g. Lambrechts and Dos Santos, 2000, Lafuma *et al*., 2001; but see Mennerat *et al*., 2008).

Finally, we did not find associations between the final plant dry mass (i.e. the dry mass of all the plant fragments extracted from the nest material after fledging) and the occurrence/abundance of blood parasites or ectoparasites. Blue tits differ from other bird species (e.g. starlings), as females incorporate fresh plant fragments while eliminating older ones (Lambrechts and Dos Santos, 2000; Petit *et al*., 2002). Thus, final dry mass represents the total mass of plants present during the nestling period, which might not be necessarily the same at previous stages of reproduction. It is possible, however, that plant provisioning during the nestling period does affect the immune status of the nestlings and not of the females. Therefore, additional investigations on offspring health will be necessary to understand plant provisioning behaviour at the end of reproduction.

## Conclusions

Our results show that the provisioning of specific secondary plants by blue tit females was in part associated with their infection status by haemosporidian parasites (i.e. *Plasmodium*, *Haemoproteus*, *Leucocytozoon*), but that such associations changed throughout the breeding season. In particular, females that had provided fewer plant fragments before incubation were more likely to be infected by *Plasmodium*. Moreover, females that had provided *Achillea* sp. fragments at early stages of reproduction were less likely to be infected by both *Plasmodium* and *Haemoproteus*. Thus, secondary plants may create an early nest environment that may repel the blood-parasite vectors (‘Nest protection hypothesis’) or directly favour that females cope with infection (‘Drug hypothesis’). However, contrary to our predictions, *Leucocytozoon* infection was positively related to the presence of other plant species (*Teucrium* sp. and *Lavandula* sp.). Due to the frequent parent-offspring transmission of *Leucozytozoon* in avian nests, but not of the other 2 haemoparasites, it is possible that females already infected by *Leucozytozoon* increased plant provisioning to prevent such transmission or as a self-medication strategy. Our study provided indirect evidence both for the ‘Nest protection hypothesis’ and the ‘Drug hypothesis’, as we did not measure vector numbers in nests (Tomás *et al*., 2008) nor the immune system or other physiological responses of blue tit females (Morales *et al*., 2004). Due to the correlative nature of this study, we cannot discern the causal role of the associations that we found. However, to our knowledge, this study constitutes the first approximation to exploring the link between plant provisioning behaviour and the blood-parasite status of the females, which are the plant gatherers in this bird species. We hope that this study serves as a starting point for future experimental research in this topic.

## Supporting information

García-Campa et al. supplementary material García-Campa et al. supplementary material

## Data Availability

García-Campa, Jorge; González-Braojos, Sonia; Morales, Judith (2024). Nest secondary plants and their associations with haemosporidian blood parasites in blue tit females. figshare. Dataset. https://doi.org/10.6084/m9.figshare.26348296.v1

## Supplementary material

The supplementary material for this article can be found at https://doi.org/10.1017/S0031182024000775.
