# Supplementary material for: Nest secondary plants and their associations with haemosporidian blood parasites in blue tit females
Source: Parasitology. 2024 Nov 12;151(10):1126–36. doi: 10.1017/S0031182024000775 (PMC11894026; doi:10.1017/S0031182024000775)
Supplement: García-Campa et al. supplementary material [file S0031182024000775sup001.docx]

Nest secondary plants and their associations with haemosporidian blood parasites in blue tit females

Jorge García-Campa^a, b *^, Sonia González-Braojos^a,c^ & Judith Morales^a^

^a^ Department of Evolutionary Ecology, National Museum of Natural Sciences – Spanish National Research Council (CSIC). c/ José Gutiérrez Abascal 2, 28006 Madrid, Spain.

^b^ CIBIO-InBIO, Universidade do Porto, Campus de Vairão, Rua Padre Armando Quintas, 4485-661 Vairão, Portugal.

^c^ Instituto de Salud Carlos III. Carretera de Majadahonda - Pozuelo, Km. 2.200, 28220, Majadahonda (Madrid), Spain.

*Correspondence: [jgarciacampa@gmail.com](mailto:jgarciacampa@gmail.com)

*Supplementary General procedures*

During the breeding season, we performed a parallel study, in which we placed feeders inside nest-boxes to supplement females with carotenoids (mainly lutein mixed with fat) prior and during egg laying (for details, see García-Campa *et al*. 2020). In brief, lutein was provided every second day until the onset of incubation, and each dosage consisted of 50 mg of Versele Laga Yel-lux Oropharma (lutein 8,000 mg/kg), which corresponds to 0.4 mg of lutein. Each lutein dose was mixed with 5 g of commercial fat with nuts (GRANA Oryx). Control nests received the same amount of fat but without lutein.

| **Species** | **Before incubation**  ***Vs***  **Incubation** | **Incubation**  ***Vs***  **Nestling period** | **Before incubation**  ***Vs***  **Nestling period** |
| --- | --- | --- | --- |
|  |  |  |  |
| *Lavandula* sp. | *X^2^* _(1, 67_) = 1.05  *P* = 0.31 | *X^2^* _(1, 67_) = 15.39  ***P* < 0.0001** | *X^2^* _(1, 66_) =  ***P* < 0.0001** |
| *Anthriscus* sp. | *X^2^* _(1, 67_) = 4.30  ***P* = 0.038** | *X^2^* _(1, 67_) = 3.75  *P* = 0.053 | *X^2^* _(1, 66_) = 0.02  *P* = 0.89 |
| *Thymus* sp | *X^2^* _(1, 67_) = 0.15  *P* = 0.70 | *X^2^* _(1, 67_) = 14.55  ***P* < 0.0001** | *X^2^* _(1, 66_) = 16.81  ***P* < 0.0001** |
| *Achillea* sp. | *X^2^* _(1, 67_) = 0.18  *P* = 0.69 | *X^2^* _(1, 67_) = 0.81  *P* = 0.37 | *X^2^* _(1, 66_) = 0.22  *P* = 0.64 |
| *Teucrium* sp. | *X^2^* _(1, 67_) = 0.18  *P* = 0.67 | *X^2^* _(1, 67_) = 0.0001  *P* =0.98 | *X^2^* _(1, 66_) = 0.09  *P* =0.77 |
| *Lamium* sp. | *X^2^* _(1, 67_) = 0.61  *P* = 0.44 | *X^2^* _(1, 67_) = 1.67  *P* = 0.20 | *X^2^* _(1, 66_) = 4.06  ***P* = 0.044** |
| *Clinopodium* sp. | *X^2^* _(1, 67_) = 0.34  *P* = 0.56 | *X^2^* _(1, 67_) = 0.58  *P* = 0.45 | *X^2^* _(1, 66_) = 2.19  *P* = 0.14 |
|  |  |  |  |

**Table S1.** Chi-square tests comparing the occurrence of the seven plant genera (*Lavandula* sp., *Anthriscus* sp., *Thymus* sp., *Achillea* sp., *Teucrium* sp., *Lamium* sp., and *Clinopodium* sp.) between the different periods: i) before incubation *Vs* incubation, ii) incubation *Vs* nestling period, and iii) before incubation *Vs* nestling period). Significant results are marked in bold (*P* < 0.05). See also Table 1, for the percentages in the different periods.

**Table S2.** Final Generalized linear models (GLMs) with binomial error distribution and negative binomial distribution (i.e., *Protocalliphora* abundance) showing the associations of the different plant variables regardless of plant genera (i.e., final plant dry mass and mean number of fragments during three reproductive stages) with the females’ haemosporidian infection (i.e., *Plasmodium*, *Haemoproteus* and *Leucocytozoon*) and with nest-dwelling ectoparasites (i.e., *Protocalliphora* and *Dermanyssus*). This table includes the full models with all predictor variables (i.e., including lutein treatment, female mass and laying date). Coefficients are shown for lutein nests and significant effects (*P* < 0.05) are marked in bold.

|  |  | ***Plasmodium*** | ***Haemoproteus*** | ***Leucocytozoon*** | ***Protocalliphora***  **(presence/absence)** | | ***Protocalliphora***  **(number)** | ***Dermanyssus*** |
| --- | --- | --- | --- | --- | --- | --- | --- | --- |
| **PRE-INCUBATION** | *(Intercept)* | *coef.* = -3.64 ± 6.05 *P* = 0.55 | *coef.* = 1.07 ± 5.99 *P* = 0.86 | *coef.* = 3.05 ± 7.00 *P* = 0.66 | *coef.* = -1.09 ± 6.35 *P* = 0.86 | | *coef.* = -1.30 ± 5.28  *P* = 0.81 | *coef.* = -4.38 ± 6.71 *P* = 0.51 |
|  | ***Mean of plants*** | *χ²₁* = 4.92 *coef.* = -1.10 ± 0.53 ***P* = 0.039** | *χ²₁* = 3.28 *coef.* = -0.87 ± 0.50 *P* = 0.080 | *χ²₁* = 0.31 *coef.* = 0.28 ± 0.50 *P* = 0.57 | *χ²₁* = 3.48 *coef.* = -0.92 ± 0.52 *P* = 0.076 | | *χ²₁* = 2.87 *coef.* = -0.95 ± 0.44 ***P* = 0.030** | *χ²₁* = 1.60 *coef.* = -0.75 ± 0.64 *P* = 0.24 |
|  | ***Lutein*** | *χ²₁* = 0.09 *coef.* = 0.08 ± 0.28  *P* = 0.77 | *χ²₁* = 0.26 *coef.* = 0.14 ± 0.28 *P* = 0.61 | *χ²₁* = 1.74 *coef.* = 0.46 ± 0.36 *P* = 0.21 | *χ²₁* = 2.14 *coef.* = -0.42 ± 0.29  *P* = 0.15 | | *χ²₁* = 0.62 *coef.* = -0.19 ± 0.24  *P* =0.44 | *χ²₁* = 0.90 *coef.* = 0.30 ± 0.32  *P* = 0.35 |
|  | ***Female mass*** | *χ²₁* = 0.84 *coef.* = 0.52 ± 0.57 *P* = 0.37 | *χ²₁* = 0.02 *coef. =* 0.08 ± 0.56 *P* = 0.89 | *χ²₁* = 0.17 *coef.* = -0.26 ± 0.65 *P* = 0.69 | *χ²₁* = 0.27 *coef.* = 0.31 ± 0.60 *P* = 0.60 | | *χ²₁* = 0.30 *coef.* = 0.31 ± 0.50 *P* = 0.54 | χ²₁ = 0.24 *coef.* = 0.31 ± 0.64 *P* = 0.62 |
|  | ***Laying date*** | *χ²₁* = 0.62 *coef.* = -0.07 ± 0.09 *P* = 0.44 | *χ²₁* =0.88 *coef.* = -0.08 ± 0.09 *P* = 0.35 | *χ²₁* = 2.04 *coef.* = -0.16 ± 0.11 *P* = 0.16 | *χ²₁* = 1.13 *coef.* = -0.10 ± 0.09 *P* = 0.29 | | *χ²₁* = 0.06 *coef.* = 0.02 ± 0.08 *P* = 0.79 | *χ²₁ =* 0.11 *coef*. = 0.03 ± 0.10 *P* = 0.74 |
|  |  |  |  |  |  | |  |  |
| **INCUBATION** | *(Intercept)* | *coef.* = -5.72 ± 5.92 *P* = 0.33 | *coef.* = -1.29 ± 5.92 *P* = 0.83 | *coef.* = 3.42 ± 6.91 *P* = 0.62 | *coef.* = -2.04 ± 6.22 *P* = 0.74 | | *coef.* = 0.01 ± 5.45 *P* = 0.99 | *coef.* = -5.39 ± 6.75 *P* = 0.42 |
|  | ***Mean of plants*** | *χ²₁* = 0.71 *coef.* = -0.17 ± 0.20 *P* = 0.41 | *χ²₁* = 0.99 *coef.* = -0.20 ± 0.20 *P* = 0.32 | *χ²₁* = 0.39 *coef.* = -0.15 ± 0.25 *P* = 0.55 | *χ²₁* = 0.05 *coef.* = 0.05 ± 0.21 *P* = 0.82 | | *χ²₁* = 0.0003 *coef.* = 0.004 ± 0.18 *P* = 0.98 | *χ²₁* = 0.82 *coef.* = -0.05 ± 0.23 *P* = 0.82 |
|  | ***Lutein*** | *χ²₁* = 0.01 *coef.* = 0.03 ± 0.27 *P* = 0.92 | *χ²₁* = 0.12 *coef.* = 0.10 ± 0.27  *P* = 0.73 | *χ²₁* = 2.10 *coef.* = 0.50 ± 0.37  *P* = 0.17 | *χ²₁* = 2.71 *coef.* = -0.46 ± 0.29  *P* = 0.11 | | *χ²₁ =* 0.69 *coef.* = -0.20 *±* 0.25 *P* = 0.41 | *χ²₁* = 0.41 *coef.* = 0.26 ± 0.32  *P* = 0.42 |
|  | ***Female mass*** | *χ²₁* = 1.37 *coef.* = 0.65 ± 0.57 *P* = 0.25 | *χ²₁* = 0.22 *coef.* = 0.27 ± 0.57 *P* = 0.64 | *χ²₁* = 0.15 *coef.* = -0.25 ± 0.65 *P* = 0.70 | *χ²₁* = 0.26 *coef.* = 0.30 ± 0.60 *P* = 0.61 | | *χ²₁* = 0.05 *coef.* = 0.14 ± 0.53 *P* = 0.79 | *χ²₁* = 0.57 *coef.* = 0.36 ± 0.65 *P* = 0.57 |
|  | ***Laying date*** | *χ²₁* = 0.16 *coef.* = -0.03 ± 0.08 *P* = 0.69 | *χ²₁* = 0.44 *coef.* = -0.06 ± 0.08 *P* = 0.51 | *χ²₁* = 2.72 *coef.* = -0.18 ± 0.11 *P* = 0.11 | *χ²₁* = 0.32 *coef.* = -0.05 ± 0.09 *P* = 0.57 | | *χ²₁* = 0.07 *coef.* = 0.02 ± 0.08 *P* = 0.79 | *χ²₁* = 0.57 *coef.* = 0.05 ± 0.10 *P* = 0.57 |
|  |  |  |  |  |  |  | |  |

|  |  | ***Plasmodium*** | ***Haemoproteus*** | ***Leucocytozoon*** | ***Protocalliphora***  **(presence/abundance)** | ***Protocalliphora***  **(number)** | ***Dermanyssus*** |
| --- | --- | --- | --- | --- | --- | --- | --- |
| **NESTLING** | *(Intercept)* | *coef.* = -4.61 ± 5.86 *P* = 0.43 | *coef.* = -1.02 ± 5.86 *P* = 0.86 | *coef.* = 3.20 ± 6.94 *P* = 0.65 | *coef.* = -2.64 ± 6.31 *P* = 0.68 | *coef.* = -0.97 ± 5.44 *P* = 0.86 | *coef.* = -5.23 ± 6.75 *P* = 0.44 |
|  | ***Mean of plants*** | *χ²₁* = 0.03 *coef.* = 0.01 ± 0.30 *P* = 0.86 | *χ²₁* = 0.12 *coef.* = -0.01 ± 0.03 *P* = 0.73 | *χ²₁* = 0.08 *coef.* = -0.01 ± 0.03 *P* = 0.78 | *χ²₁* = 0.10 *coef.* = -0.01 ± 0.03 *P* = 0.76 | *χ²₁* = 0.24 *coef.* = -0.02 ± 0.03 *P* = 0.49 | *χ²₁* = 0.004 *coef.* = -0.002 ± 0.03 *P* = 0.95 |
|  | ***Lutein*** | *χ²₁* = 0.03 *coef.* = -0.04 ± 0.27  *P* = 0.87 | *χ²₁* = 0.19 *coef.* = 0.12 ± 0.27 *P* = 0.66 | *χ²₁* = 1.73 *coef.* = 0.46 ± 0.36  *P* = 0.21 | *χ²₁* = 2.62 *coef.* = -0.45 ± 0.29  *P* = 0.11 | *χ²₁ =* 0.46 *coef. =* -0.17 ± 0.24 *P =* 0.49 | *χ²₁* = 0.67 *coef.* = 0.25 ± 0.32  *P* = 0.42 |
|  | ***Female mass*** | *χ²₁* = 0.87 *coef.* = 0.51 ± 0.56 *P* = 0.36 | *χ²₁* = 0.14 *coef.* = 0.20 ± 0.56 *P* = 0.72 | *χ²₁* = 0.14 *coef.* = -0.24 ± 0.66 *P* = 0.71 | *χ²₁* = 0.40 *coef.* = 0.38 ± 0.61 *P* = 0.53 | *χ²₁* = 0.15 *coef.* = 0.24 ± 0.53 *P* = 0.65 | *χ²₁* = 0.28 *coef.* = 0.34 ± 0.64 *P* = 0.60 |
|  | ***Laying date*** | *χ²₁* = 0.10 *coef.* = -0.03 ± 0.08 *P* = 0.76 | *χ²₁* = 0.27 *coef.* = -0.04 ± 0.08 *P* = 0.61 | *χ²₁* = 2.50 *coef.* = -0.17 ± 0.11 *P* = 0.13 | *χ²₁* = 0.41 *coef.* = -0.05 ± 0.09 *P* = 0.52 | *χ²₁* = 0.14 *coef.* = 0.03 ± 0.08 *P* = 0.69 | *χ²₁* = 0.37 *coef.* = 0.06 ± 0.09 *P* = 0.55 |
|  |  |  |  |  |  |  |  |
| **FINAL PLANT**  **DRY MASS** | *(Intercept)* | *coef.* = -5.98 ± 6.42 *P* = 0.35 | *coef.* = 0.11 ± 6.24 *P* = 0.99 | *coef.* = -0.19 ± 7.45 *P* = 0.98 | *coef.* = -1.34 ± 6.37 *P* = 0.83 | *coef.* = 2.00 ± 5.56  *P* = 0.72 | *coef.* = -7.45 ± 6.91 *P* = 0.28 |
|  | ***Plant dry mass*** | *χ²₁* = 1.46 *coef.* = 0.01 ± 0.01 *P* = 0.24 | *χ²₁* = 0.03 *coef.* = 0.002 ± 0.01 *P* = 0.86 | *χ²₁* = 0.59 *coef.* = 0.01 ± 0.01 *P* = 0.44 | *χ²₁* = 0.27 *coef.* = -0.01 ± 0.01 *P* = 0.60 | *χ²₁* = 0.77 *coef.* = -0.01 ± 0.01 *P* = 0.33 | *χ²₁* = 1.52 *coef.* = 0.01 ± 0.01 *P* = 0.21 |
|  | ***Lutein*** | *χ²₁* = 0.13 *coef.* = -0.10 ± 0.28  *P* = 0.72 | *χ²₁* = 0.12 *coef.* = 0.10 ± 0.27  *P* = 0.73 | *χ²₁* = 1.55 *coef.* = 0.44 ± 0.37  *P* = 0.23 | *χ²₁* = 2.37 *coef.* = -0.43 ± 0.29  *P* = 0.13 | *χ²₁ =* 0.69 *coef. =* -0.20 ± 0.25 *P =* 0.42 | *χ²₁* = 0.36 *coef.* = 0.19 ± 0.32  *P* = 0.56 |
|  | ***Female mass*** | *χ²₁* = 1.10 *coef.* = 0.63 ± 0.61 *P* = 0.30 | *χ²₁* = 0.01 *coef.* = 0.07 ± 0.59 *P* = 0.91 | *χ²₁* = 0.02 *coef.* = 0.10 ± 0.71 *P* = 0.90 | *χ²₁* = 0.18 *coef.* = 0.25 ± 0.61 *P* = 0.68 | *χ²₁* = 0.0002 *coef.* = -0.01 ± 0.53 *P* = 0.99 | *χ²₁* = 0.70 *coef.* = 0.54 ± 0.65 *P* = 0.41 |
|  | ***Laying date*** | *χ²₁* = 0.05 *coef.* = -0.02 ± 0.08 *P* = 0.82 | *χ²₁* = 0.17 *coef.* = -0.03 ± 0.08 *P* = 0.68 | *χ²₁* = 2.78 *coef.* = -0.18 ± 0.11 *P* = 0.11 | *χ²₁* = 0.40 *coef.* = -0.05 ± 0.09 *P* = 0.53 | *χ²₁* = 0.01 *coef.* = -0.01 ± 0.07 *P* = 0.92 | *χ²₁* = 0.40 *coef.* = 0.06 ± 0.09 *P* = 0.53 |
|  |  |  |  |  |  |  |  |

**Table S3.** Final Generalized linear models (GLMs) with binomial error showing the effects of the presence of green plants per plant species (a) *Achillea* sp., b) *Lavandula* sp., c) *Anthriscus* sp., d) *Teucrium* sp., e) *Thymus* sp., f) *Lamium* sp. on blood parasite occurrence (i.e., *Plasmodium*, *Haemoproteus* and *Leucocytozoon*) and nest-dwelling ectoparasites (i.e., *Protocalliphora* and *Dermanyssus*) provided by blue tit females before the incubation, incubation and nestling period. These tables include the full models show in Table 2, but here including all predictor variables (i.e., female mass and laying date). Significant effects (P < 0.05) are marked in bold. Models for the abundance of *Protocalliphora* blowflies are shown in Table S5.

| 1. ***Achillea* sp. before incubation** | | | | | | | | | | | | | | | | | | | | | | | | | | | | | | | | | | | | | | | | | | |
| --- | --- | --- | --- | --- | --- | --- | --- | --- | --- | --- | --- | --- | --- | --- | --- | --- | --- | --- | --- | --- | --- | --- | --- | --- | --- | --- | --- | --- | --- | --- | --- | --- | --- | --- | --- | --- | --- | --- | --- | --- | --- | --- |
|  | ***Plasmodium*** | | | | | | | | ***Haemoproteus*** | | | | | | | | ***Leucocytozoon*** | | | | | | | | ***Protocalliphora*** | | | | | | | | | | | | | | ***Dermanyssus*** | | | |
|  | | | | |  | | | | | | | | |  | | | | | | | |  | | | | | | | |  | | | | | | | | |  | | | |
| *Predictors* | *Chis SQ* | *Log-Odds* | | *std. Error* | | | *p* | *Chis SQ* | | | | | *Log-Odds* | *std. Error* | | *p* | | | | *Chis SQ* | *Log-Odds* | | *std. Error* | | *p* | | | *Chis SQ* | | *Log-Odds* | *std. Error* | | *p* | *Chis SQ* | | | | | | *Log-Odds* | *std. Error* | *p* |
| (Intercept) |  | -4.58 | | 6.13 | | | 0.455 |  | | | | | 0.42 | 6.14 | | 0.946 | | | |  | 2.13 | | 6.74 | | 0.753 | | |  | | -0.65 | 6.17 | | 0.916 |  | | | | | | -5.77 | 6.69 | 0.389 |
| *Achillea* sp. presence | 5.73 | -1.82 | | 0.85 | | | **0.031** | 4.79 | | | | | -1.58 | 0.76 | | **0.037** | | | | 1.23 | -1.11 | | 1.12 | | 0.32 | | | 2.61 | | -1.20 | 0.77 | | 0.118 | 1.22 | | | | | | -1.10 | 1.11 | 0.323 |
| Female mass | 0.99 | 0.57 | | 0.58 | | | 0.331 | 0.05 | | | | | 0.13 | 0.58 | | 0.829 | | | | 0.01 | -0.05 | | 0.63 | | 0.936 | | | 0.06 | | 0.15 | 0.59 | | 0.80 | 0.53 | | | | | | 0.46 | 0.64 | 0.468 |
| Laying date | 0.39 | -0.05 | | 0.08 | | | 0.533 | 0.78 | | | | | -0.08 | 0.09 | | 0.38 | | | | 4.54 | -0.22 | | 0.11 | | **0.047** | | | 0.10 | | -0.03 | 0.08 | | 0.751 | 0.02 | | | | | | 0.01 | 0.09 | 0.885 |
| Observations | | |  | | | 64 | |  | | | | | | | 64 | | | | |  | | | | 64 | | |  | | | | | 62 | |  | | | | | | | 62 | |
| R^2^ Tjur | | |  | | | 0.114 | |  | | | | | | | 0.088 | | | | |  | | | | 0.084 | | |  | | | | | 0.045 | |  | | | | | | | 0.028 | |
|  |  |  | |  | | |  |  | | | | |  |  | |  | | | |  |  | |  | |  | | |  | |  |  | |  |  | | | | | |  |  |  |
|  |  |  | |  | | |  |  | | | | |  |  | |  | | | |  |  | |  | |  | | |  | |  |  | |  |  | | | | | |  |  |  |
| 1. ***Achillea* sp. incubation** | | | | | | | | | | | | | | | | | | | | | | | | | | | | | | | | | | | | | | | | | | |
|  | ***Plasmodium*** | | | | | | | | ***Haemoproteus*** | | | | | | | | ***Leucocytozoon*** | | | | | | | | ***Protocalliphora*** | | | | | | | | | | | | | | ***Dermanyssus*** | | | |
| *Predictors* | *Chis SQ* | *Log-Odds* | | *std. Error* | | | *p* | *Chis SQ* | | | | | *Log-Odds* | *std. Error* | | *p* | | | | *Chis SQ* | *Log-Odds* | | *std. Error* | | *p* | | | *Chis SQ* | | *Log-Odds* | *std. Error* | | *p* | *Chis SQ* | | | | | | *Log-Odds* | *std. Error* | *p* |
| (Intercept) |  | -5.76 | | 5.96 | | | 0.334 |  | | | | | -0.68 | 5.84 | | 0.907 | | | |  | 2.88 | | 6.70 | | 0.668 | | |  | | -2.50 | 5.98 | | 0.676 |  | | | | | | -4.98 | 6.58 | 0.449 |
| *Achillea* sp. presence | 4.60 | -1.51 | | 0.75 | | | **0.044** | 1.45 | | | | | -0.81 | 0.67 | | 0.23 | | | | 0.13 | -0.30 | | 0.86 | | 0.728 | | | 0.20 | | 0.30 | 0.69 | | 0.659 | 0.61 | | | | | | -0.63 | 0.84 | 0.456 |
| Female mass | 1.36 | 0.65 | | 0.57 | | | 0.252 | 0.12 | | | | | 0.19 | 0.55 | | 0.731 | | | | 0.09 | -0.18 | | 0.63 | | 0.77 | | | 0.29 | | 0.31 | 0.57 | | 0.591 | 0.32 | | | | | | 0.35 | 0.63 | 0.574 |
| Laying date | 0.07 | -0.02 | | 0.08 | | | 0.786 | 0.33 | | | | | -0.05 | 0.08 | | 0.568 | | | | 3.05 | -0.17 | | 0.10 | | 0.10 | | | 0.11 | | -0.03 | 0.08 | | 0.738 | 0.21 | | | | | | 0.04 | 0.09 | 0.647 |
| Observations | | |  | | | 65 | |  | | | | | | | 65 | | | | |  | | | | 65 | | |  | | | | | 63 | |  | | | | | | | 63 | |
| R^2^ Tjur | | |  | | | 0.088 | |  | | | | | | | 0.031 | | | | |  | | | | 0.051 | | |  | | | | | 0.01 | |  | | | | | | | 0.017 | |
|  |  |  | |  | | |  |  | | | | |  |  | |  | | | |  |  | |  | |  | | |  | |  |  | |  |  | | | | | |  |  |  |
|  |  |  | |  | | |  |  | | | | |  |  | |  | | | |  |  | |  | |  | | |  | |  |  | |  |  | | | | | |  |  |  |
| 1. ***Achillea* sp. nestling** | | | | | | | | | | | | | | | | | | | | | | | | | | | | | | | | | | | | | | | | | | |
|  | ***Plasmodium*** | | | | | | | | ***Haemoproteus*** | | | | | | | | ***Leucocytozoon*** | | | | | | | | ***Protocalliphora*** | | | | | | | | | | | | | | ***Dermanyssus*** | | | |
| *Predictors* | *Chis SQ* | *Log-Odds* | | *std. Error* | | | *p* | *Chis SQ* | | | | | *Log-Odds* | *std. Error* | | *p* | | | | *Chis SQ* | *Log-Odds* | | *std. Error* | | *p* | | | *Chis SQ* | | *Log-Odds* | *std. Error* | | *p* | *Chis SQ* | | | | | | *Log-Odds* | *std. Error* | *p* |
| (Intercept) |  | -4.91 | | 5.83 | | | 0.40 |  | | | | | -0.65 | 5.76 | | 0.911 | | | |  | 1.86 | | 6.91 | | 0.788 | | |  | | -2.65 | 6.00 | | 0.658 |  | | | | | | -3.44 | 6.53 | 0.60 |
| *Achillea* sp. presence | 1.42 | -0.93 | | 0.80 | | | 0.24 | 0.01 | | | | | 0.06 | 0.78 | | 0.939 | | | | 4.73 | -17.59 | | 2252.54 | | 0.994 | | | 0.01 | | 0.06 | 0.82 | | 0.941 | 4.63 | | | | | | -16.72 | 1493.68 | 0.991 |
| Female mass | 1.06 | 0.56 | | 0.55 | | | 0.31 | 0.10 | | | | | 0.17 | 0.54 | | 0.757 | | | | 0.01 | -0.06 | | 0.66 | | 0.925 | | | 0.33 | | 0.33 | 0.57 | | 0.568 | 0.10 | | | | | | 0.20 | 0.63 | 0.748 |
| Laying date | 0.10 | -0.03 | | 0.08 | | | 0.748 | 0.33 | | | | | -0.05 | 0.08 | | 0.565 | | | | 3.19 | -0.18 | | 0.10 | | 0.09 | | | 0.11 | | -0.03 | 0.08 | | 0.74 | 0.25 | | | | | | 0.05 | 0.09 | 0.616 |
| Observations | | |  | | | 64 | |  | | | | | | | 64 | | | | |  | | | | 64 | | |  | | | | | 63 | |  | | | | | | | 63 | |
| R^2^ Tjur | | |  | | | 0.041 | |  | | | | | | | 0.008 | | | | |  | | | | 0.098 | | |  | | | | | 0.008 | |  | | | | | | | 0.052 | |
|  |  |  | |  | | |  |  | | | | |  |  | |  | | | |  |  | |  | |  | | |  | |  |  | |  |  | | | | | |  |  |  |
|  |  |  | |  | | |  |  | | | | |  |  | |  | | | |  |  | |  | |  | | |  | |  |  | |  |  | | | | | |  |  |  |
| 1. ***Lavandula* sp. before incubation** | | | | | | | | | | | | | | | | | | | | | | | | | | | | | | | | | | | | | | | | | | |
|  | ***Plasmodium*** | | | | | | | | ***Haemoproteus*** | | | | | | | | ***Leucocytozoon*** | | | | | | | | ***Protocalliphora*** | | | | | | | | | | | | | ***Dermanyssus*** | | | | |
| *Predictors* | *Chis SQ* | *Log-Odds* | | *std. Error* | | | *p* | *Chis SQ* | | | | | *Log-Odds* | *std. Error* | | *p* | | | | *Chis SQ* | *Log-Odds* | | *std. Error* | | *p* | | | *Chis SQ* | | *Log-Odds* | *std. Error* | | *p* | *Chis SQ* | | | | | | *Log-Odds* | *std. Error* | *p* |
| (Intercept) |  | -5.04 | | 5.87 | | | 0.39 |  | | | | | -0.45 | 5.78 | | 0.938 | | | |  | 2.94 | | 6.72 | | 0.661 | | |  | | -2.59 | 5.96 | | 0.664 |  | | | | | | -4.86 | 6.60 | 0.462 |
| *Lavandula* sp. presence | 0.76 | -0.56 | | 0.64 | | | 0.384 | 0.01 | | | | | -0.07 | 0.65 | | 0.916 | | | | 0.21 | 0.33 | | 0.72 | | 0.644 | | | 0.07 | | 0.17 | 0.65 | | 0.793 | 1.19 | | | | | | -0.86 | 0.84 | 0.306 |
| Female mass | 1.12 | 0.58 | | 0.56 | | | 0.30 | 0.08 | | | | | 0.16 | 0.55 | | 0.775 | | | | 0.11 | -0.21 | | 0.63 | | 0.741 | | | 0.31 | | 0.31 | 0.57 | | 0.583 | 0.33 | | | | | | 0.36 | 0.63 | 0.564 |
| Laying date | 0.17 | -0.03 | | 0.08 | | | 0.681 | 0.35 | | | | | -0.05 | 0.08 | | 0.554 | | | | 2.80 | -0.17 | | 0.11 | | 0.11 | | | 0.08 | | -0.02 | 0.08 | | 0.774 | 0.09 | | | | | | 0.03 | 0.09 | 0.765 |
| Observations | | |  | | | 65 | |  | | | | | | | 65 | | | | |  | | | | 65 | | |  | | | | | 63 | |  | | | | | | | 63 | |
| R^2^ Tjur | | |  | | | 0.03 | |  | | | | | | | 0.008 | | | | |  | | | | 0.052 | | |  | | | | | 0.009 | |  | | | | | | | 0.026 | |
|  |  |  | |  | | |  |  | | | | |  |  | |  | | | |  |  | |  | |  | | |  | |  |  | |  |  | | | | | |  |  |  |
|  |  |  | |  | | |  |  | | | | |  |  | |  | | | |  |  | |  | |  | | |  | |  |  | |  |  | | | | | |  |  |  |
| 1. ***Lavandula* sp. incubation** | | | | | | | | | | | | | | | | | | | | | | | | | | | | | | | | | | | | | | | | | | |
|  | ***Plasmodium*** | | | | | | | | ***Haemoproteus*** | | | | | | | | ***Leucocytozoon*** | | | | | | | | ***Protocalliphora*** | | | | | | | | | | | | | ***Dermanyssus*** | | | | |
| *Predictors* | *Chis SQ* | *Log-Odds* | | *std. Error* | | | *p* | *Chis SQ* | | | | | *Log-Odds* | *std. Error* | | *p* | | | | *Chis SQ* | *Log-Odds* | | *std. Error* | | *p* | | | *Chis SQ* | | *Log-Odds* | *std. Error* | | *p* | *Chis SQ* | | | | | | *Log-Odds* | *std. Error* | *p* |
| (Intercept) |  | -4.62 | | 5.99 | | | 0.441 |  | | | | | -3.98 | 6.36 | | 0.531 | | | |  | 4.03 | | 6.96 | | 0.563 | | |  | | -1.08 | 6.26 | | 0.863 |  | | | | | | -6.32 | 6.91 | 0.36 |
| *Lavandula* sp. presence | 0.07 | 0.16 | | 0.60 | | | 0.794 | 3.88 | | | | | -1.19 | 0.62 | | 0.054 | | | | 0.36 | 0.41 | | 0.68 | | 0.545 | | | 0.65 | | 0.49 | 0.62 | | 0.43 | 0.55 | | | | | | -0.51 | 0.70 | 0.466 |
| Female mass | 0.81 | 0.51 | | 0.57 | | | 0.376 | 0.80 | | | | | 0.54 | 0.62 | | 0.379 | | | | 0.23 | -0.31 | | 0.66 | | 0.639 | | | 0.07 | | 0.16 | 0.61 | | 0.80 | 0.54 | | | | | | 0.49 | 0.66 | 0.462 |
| Laying date | 0.09 | -0.02 | | 0.08 | | | 0.77 | 0.34 | | | | | -0.05 | 0.08 | | 0.559 | | | | 3.14 | -0.18 | | 0.11 | | 0.091 | | | 0.10 | | -0.03 | 0.08 | | 0.749 | 0.21 | | | | | | 0.04 | 0.09 | 0.647 |
| Observations | | |  | | | 65 | |  | | | | | | | 65 | | | | |  | | | | 65 | | |  | | | | | 63 | |  | | | | | | | 63 | |
| R^2^ Tjur | | |  | | | 0.02 | |  | | | | | | | 0.066 | | | | |  | | | | 0.053 | | |  | | | | | 0.017 | |  | | | | | | | 0.015 | |
|  |  |  | |  | | |  |  | | | | |  |  | |  | | | |  |  | |  | |  | | |  | |  |  | |  |  | | | | | |  |  |  |
|  |  |  | |  | | |  |  | | | | |  |  | |  | | | |  |  | |  | |  | | |  | |  |  | |  |  | | | | | |  |  |  |
| ***b) Lavandula* sp. nestling** | | | | | | | | | | | | | | | | | | | | | | | | | | | | | | | | | | | | | | | | | | |
|  | ***Plasmodium*** | | | | | | | | ***Haemoproteus*** | | | | | | | | ***Leucocytozoon*** | | | | | | | | ***Protocalliphora*** | | | | | | | | | | | | | ***Dermanyssus*** | | | | |
| *Predictors* | *Chis SQ* | *Log-Odds* | | *std. Error* | | | *p* | *Chis SQ* | | | | | *Log-Odds* | *std. Error* | | *p* | | | | *Chis SQ* | *Log-Odds* | | *std. Error* | | *p* | | | *Chis SQ* | | *Log-Odds* | *std. Error* | | *p* | *Chis SQ* | | | | | | *Log-Odds* | *std. Error* | *p* |
| (Intercept) |  | -4.49 | | 5.8 | | | 0.439 |  | | | | | -0.80 | 5.83 | | 0.891 | | | |  | 4.98 | | 8.05 | | 0.536 | | |  | | -2.42 | 5.99 | | 0.686 |  | | | | | | -4.10 | 6.94 | 0.555 |
| *Lavandula* sp. presence | 0.95 | 0.51 | | 0.53 | | | 0.332 | 0.19 | | | | | -0.23 | 0.54 | | 0.664 | | | | 6.75 | 1.91 | | 0.85 | | **0.025** | | | 0.07 | | 0.14 | 0.53 | | 0.786 | 2.05 | | | | | | 0.90 | 0.65 | 0.169 |
| Female mass | 0.77 | 0.48 | | 0.55 | | | 0.387 | 0.13 | | | | | 0.20 | 0.55 | | 0.721 | | | | 0.45 | -0.50 | | 0.77 | | 0.515 | | | 0.27 | | 0.30 | 0.58 | | 0.609 | 0.08 | | | | | | 0.19 | 0.66 | 0.772 |
| Laying date | 0.10 | -0.03 | | 0.08 | | | 0.747 | 0.32 | | | | | -0.05 | 0.08 | | 0.572 | | | | 3.65 | -0.20 | | 0.11 | | 0.069 | | | 0.11 | | -0.03 | 0.08 | | 0.741 | 0.23 | | | | | | 0.04 | 0.09 | 0.634 |
| Observations | | |  | | | 64 | |  | | | | | | | 64 | | | | |  | | | | 64 | | | | |  | | | 63 | |  | | | | | | | 63 | |
| R^2^ Tjur | | |  | | | 0.033 | |  | | | | | | | 0.011 | | | | |  | | | | 0.151 | | | | |  | | | 0.009 | |  | | | | | | | 0.038 | |
|  |  |  | |  | | |  |  | | | | |  |  | |  | | | |  |  | |  | |  | | |  | |  |  | |  |  | | | | | |  |  |  |
| 1. ***Anthriscus* sp. before incubation** | | | | | | | | | | | | | | | | | | | | | | | | | | | | | | | | | | | | | | | | | | |
|  | ***Plasmodium*** | | | | | | | | ***Haemoproteus*** | | | | | | | | ***Leucocytozoon*** | | | | | | | | ***Protocalliphora*** | | | | | | | | | | | | | ***Dermanyssus*** | | | | |
| *Predictors* | *Chis SQ* | *Log-Odds* | | *std. Error* | | | *p* | *Chis SQ* | | | | | *Log-Odds* | *std. Error* | | *p* | | | | *Chis SQ* | *Log-Odds* | | *std. Error* | | *p* | | | *Chis SQ* | | *Log-Odds* | *std. Error* | | *p* | *Chis SQ* | | | | | | *Log-Odds* | *std. Error* | *p* |
| (Intercept) |  | -5.05 | | 5.82 | | | 0.39 |  | | | | | -0.45 | 5.79 | | 0.94 | | | |  | 3.23 | | 6.77 | | 0.63 | | |  | | -2.51 | 5.98 | | 0.68 |  | | | | | | -4.98 | 6.62 | 0.45 |
| *Anthriscus* sp. presence | 0.20 | -0.25 | | 0.56 | | | 0.66 | 0.28 | | | | | -0.30 | 0.57 | | 0.60 | | | | 0.67 | 0.52 | | 0.64 | | 0.41 | | | 0.27 | | 0.30 | 0.58 | | 0.61 | 0.49 | | | | | | -0.46 | 0.67 | 0.49 |
| Female mass | 1.11 | 0.57 | | 0.55 | | | 0.30 | 0.10 | | | | | 0.17 | 0.55 | | 0.75 | | | | 0.15 | -0.25 | | 0.64 | | 0.70 | | | 0.27 | | 0.30 | 0.58 | | 0.61 | 0.34 | | | | | | 0.37 | 0.63 | 0.56 |
| Laying date | 0.13 | -0.03 | | 0.08 | | | 0.72 | 0.44 | | | | | -0.05 | 0.08 | | 0.51 | | | | 2.72 | -0.17 | | 0.11 | | 0.11 | | | 0.06 | | -0.02 | 0.08 | | 0.81 | 0.11 | | | | | | 0.03 | 0.09 | 0.74 |
| Observations | | |  | | | 65 | |  | | | | | | | 65 | | | | |  | | | | 65 | | | | |  | | | 63 | |  | | | | | | | 63 | |
| R^2^ Tjur | | |  | | | 0.022 | |  | | | | | | | 0.012 | | | | |  | | | | 0.062 | | | | |  | | | 0.012 | |  | | | | | | | 0.015 | |
|  |  |  | |  | | |  |  | | | | |  |  | |  | | | |  |  | |  | |  | | |  | |  |  | |  |  | | | | | |  |  |  |
|  |  |  | |  | | |  |  | | | | |  |  | |  | | | |  |  | |  | |  | | |  | |  |  | |  |  | | | | | |  |  |  |
| ***c) Anthriscus* sp. incubation** | | | | | | | | | | | | | | | | | | | | | | | | | | | | | | | | | | | | | | | | | | |
|  | ***Plasmodium*** | | | | | | | | ***Haemoproteus*** | | | | | | | | ***Leucocytozoon*** | | | | | | | | ***Protocalliphora*** | | | | | | | | | | | | | ***Dermanyssus*** | | | | |
| *Predictors* | *Chis SQ* | *Log-Odds* | | *std. Error* | | | *p* | *Chis SQ* | | | | | *Log-Odds* | *std. Error* | | *p* | | | | *Chis SQ* | *Log-Odds* | | *std. Error* | | *p* | | | *Chis SQ* | | *Log-Odds* | *std. Error* | | *p* | *Chis SQ* | | | | | | *Log-Odds* | *std. Error* | *p* |
| (Intercept) |  | -4.85 | | 5.84 | | | 0.41 |  | | | | | 0.57 | 5.93 | | 0.92 | | | |  | 3.08 | | 6.72 | | 0.65 | | |  | | -3.51 | 6.08 | | 0.56 |  | | | | | | -4.38 | 6.65 | 0.51 |
| *Anthriscus* sp. presence | 0.07 | 0.19 | | 0.72 | | | 0.79 | 2.02 | | | | | 1.12 | 0.85 | | 0.19 | | | | 0.003 | 0.05 | | 0.88 | | 0.96 | | | 0.79 | | -0.66 | 0.75 | | 0.38 | 0.74 | | | | | | 0.26 | 0.79 | 0.74 |
| Female mass | 0.97 | 0.54 | | 0.55 | | | 0.33 | 0.01 | | | | | 0.06 | 0.56 | | 0.92 | | | | 0.11 | -0.21 | | 0.63 | | 0.74 | | | 0.50 | | 0.41 | 0.58 | | 0.48 | 0.65 | | | | | | 0.28 | 0.63 | 0.65 |
| Laying date | 0.11 | -0.03 | | 0.08 | | | 0.75 | 0.61 | | | | | -0.06 | 0.08 | | 0.44 | | | | 3.04 | -0.18 | | 0.11 | | 0.10 | | | 0.04 | | -0.02 | 0.08 | | 0.84 | 0.68 | | | | | | 0.04 | 0.09 | 0.68 |
| Observations | | |  | | | 65 | |  | | | | | | | 65 | | | | |  | | | | 65 | | |  | | | | | 63 | |  | | | | | | | 63 | |
| R^2^ Tjur | | |  | | | 0.02 | |  | | | | | | | 0.037 | | | | |  | | | | 0.049 | | |  | | | | | 0.02 | |  | | | | | | | 0.009 | |
|  |  |  | |  | | |  |  | | | | |  |  | |  | | | |  |  | |  | |  | | |  | |  |  | |  |  | | | | | |  |  |  |
|  |  |  | |  | | |  |  | | | | |  |  | |  | | | |  |  | |  | |  | | |  | |  |  | |  |  | | | | | |  |  |  |
| ***c) Anthriscus* sp. nestling** | | | | | | | | | | | | | | | | | | | | | | | | | | | | | | | | | | | | | | | | | | |
|  | ***Plasmodium*** | | | | | | | | ***Haemoproteus*** | | | | | | | | ***Leucocytozoon*** | | | | | | | | ***Protocalliphora*** | | | | | | | | | | | | | ***Dermanyssus*** | | | | |
| *Predictors* | *Chis SQ* | *Log-Odds* | | *std. Error* | | | *p* | *Chis SQ* | | | | | *Log-Odds* | *std. Error* | | *p* | | | | *Chis SQ* | *Log-Odds* | | *std. Error* | | *p* | | | *Chis SQ* | | *Log-Odds* | *std. Error* | | *p* | *Chis SQ* | | | | | | *Log-Odds* | *std. Error* | *p* |
| (Intercept) |  | -4.58 | | 5.83 | | | 0.43 |  | | | | | -0.79 | 5.79 | | 0.89 | | | |  | 3.73 | | 6.79 | | 0.58 | | |  | | -2.96 | 6.02 | | 0.62 |  | | | | | | -4.83 | 6.59 | 0.46 |
| *Anthriscus* sp. presence | 0.40 | 0.36 | | 0.57 | | | 0.53 | 0.13 | | | | | -0.21 | 0.56 | | 0.71 | | | | 1.15 | 0.69 | | 0.64 | | 0.28 | | | 0.33 | | -0.32 | 0.56 | | 0.57 | 0.02 | | | | | | -0.08 | 0.63 | 0.89 |
| Female mass | 0.85 | 0.5 | | 0.55 | | | 0.36 | 0.12 | | | | | 0.19 | 0.55 | | 0.73 | | | | 0.20 | -0.28 | | 0.64 | | 0.66 | | | 0.41 | | 0.37 | 0.58 | | 0.52 | 0.28 | | | | | | 0.33 | 0.63 | 0.60 |
| Laying date | 0.09 | -0.02 | | 0.08 | | | 0.77 | 0.33 | | | | | -0.05 | 0.08 | | 0.56 | | | | 3.26 | -0.18 | | 0.11 | | 0.09 | | | 0.11 | | -0.03 | 0.08 | | 0.74 | 0.21 | | | | | | 0.04 | 0.09 | 0.64 |
| Observations | | |  | | | 64 | |  | | | | | | | 64 | | | | |  | | | | 64 | | | | |  | | | 63 | |  | | | | | | | 63 | |
| R^2^ Tjur | | |  | | | 0.023 | |  | | | | | | | 0.01 | | | | |  | | | | 0.063 | | | | |  | | | 0.013 | |  | | | | | | | 0.007 | |
|  |  |  | |  | | |  |  | | | | |  |  | |  | | | |  |  | |  | |  | | |  | |  |  | |  |  | | | | | |  |  |  |
| 1. ***Teucrium* sp. before incubation** | | | | | | | | | | | | | | | | | | | | | | | | | | | | | | | | | | | | | | | | | | |
|  | ***Plasmodium*** | | | | | | | | ***Haemoproteus*** | | | | | | | | ***Leucocytozoon*** | | | | | | | | ***Protocalliphora*** | | | | | | | | | | | | | ***Dermanyssus*** | | | | |
| *Predictors* | *Chis SQ* | *Log-Odds* | | *std. Error* | | | *p* | *Chis SQ* | | | | | *Log-Odds* | *std. Error* | | *p* | | | | *Chis SQ* | *Log-Odds* | | *std. Error* | | *p* | | | *Chis SQ* | | *Log-Odds* | *std. Error* | | *p* | *Chis SQ* | | | | | | *Log-Odds* | *std. Error* | *p* |
| (Intercept) |  | -4.91 | | 5.90 | | | 0.41 |  | | | | | -0.24 | 5.86 | | 0.97 | | | |  | 3.13 | | 7.08 | | 0.66 | | |  | | -2.55 | 6.09 | | 0.68 |  | | | | | | -5.08 | 6.69 | 0.45 |
| *Teucrium* sp. presence | 2.43 | -1.32 | | 0.89 | | | 0.14 | 1.21 | | | | | -0.90 | 0.82 | | 0.28 | | | | 4.04 | 1.75 | | 0.87 | | **0.05** | | | 2.78 | | -1.41 | 0.89 | | 0.12 | 1.00 | | | | | | 0.85 | 0.83 | 0.31 |
| Female mass | 1.06 | 0.57 | | 0.56 | | | 0.31 | 0.07 | | | | | 0.15 | 0.55 | | 0.79 | | | | 0.13 | -0.24 | | 0.66 | | 0.72 | | | 0.36 | | 0.35 | 0.58 | | 0.55 | 0.27 | | | | | | 0.33 | 0.63 | 0.60 |
| Laying date | 0.16 | -0.03 | | 0.08 | | | 0.69 | 0.42 | | | | | -0.05 | 0.08 | | 0.52 | | | | 2.83 | -0.17 | | 0.11 | | 0.11 | | | 0.21 | | -0.04 | 0.08 | | 0.65 | 0.30 | | | | | | 0.05 | 0.09 | 0.59 |
| Observations | | |  | | | 65 | |  | | | | | | | 65 | | | | |  | | | | 65 | | | | |  | | | 63 | |  | | | | | | | 63 | |
| R^2^ Tjur | | |  | | | 0.056 | |  | | | | | | | 0.027 | | | | |  | | | | 0.126 | | | | |  | | | 0.051 | |  | | | | | | | 0.023 | |
|  |  |  | |  | | |  |  | | | | |  |  | |  | | | |  |  | |  | |  | | |  | |  |  | |  |  | | | | | |  |  |  |
|  |  |  | |  | | |  |  | | | | |  |  | |  | | | |  |  | |  | |  | | |  | |  |  | |  |  | | | | | |  |  |  |
| ***d) Teucrium* sp. incubation** | | | | | | | | | | | | | | | | | | | | | | | | | | | | | | | | | | | | | | | | | | |
|  | ***Plasmodium*** | | | | | | | | ***Haemoproteus*** | | | | | | | | ***Leucocytozoon*** | | | | | | | | ***Protocalliphora*** | | | | | | | | | | | | | ***Dermanyssus*** | | | | |
| *Predictors* | *Chis SQ* | *Log-Odds* | | *std. Error* | | | *p* | *Chis SQ* | | | | | *Log-Odds* | *std. Error* | | *p* | | | | *Chis SQ* | *Log-Odds* | | *std. Error* | | *p* | | | *Chis SQ* | | *Log-Odds* | *std. Error* | | *p* | *Chis SQ* | | | | | | *Log-Odds* | *std. Error* | *p* |
| (Intercept) |  | -5.04 | | 5.79 | | | 0.39 |  | | | | | -0.45 | 5.79 | | 0.94 | | | |  | 3.08 | | 6.97 | | 0.66 | | |  | | -2.61 | 5.97 | | 0.66 |  | | | | | | -4.85 | 6.66 | 0.47 |
| *Teucrium* sp. presence | 0.01 | 0.11 | | 0.97 | | | 0.91 | 0.03 | | | | | -0.18 | 0.97 | | 0.85 | | | | 2.61 | 1.61 | | 1.00 | | 0.11 | | | 0.00 | | 0.04 | 0.97 | | 0.97 | 0.52 | | | | | | 0.73 | 0.98 | 0.46 |
| Female mass | 1.04 | 0.55 | | 0.55 | | | 0.32 | 0.08 | | | | | 0.16 | 0.55 | | 0.77 | | | | 0.15 | -0.25 | | 0.66 | | 0.71 | | | 0.32 | | 0.32 | 0.57 | | 0.57 | 0.24 | | | | | | 0.31 | 0.63 | 0.63 |
| Laying date | 0.07 | -0.02 | | 0.08 | | | 0.79 | 0.37 | | | | | -0.05 | 0.08 | | 0.55 | | | | 2.26 | -0.16 | | 0.11 | | 0.15 | | | 0.10 | | -0.03 | 0.08 | | 0.75 | 0.34 | | | | | | 0.05 | 0.09 | 0.56 |
| Observations | | |  | | | 65 | |  | | | | | | | 65 | | | | |  | | | | 65 | | | | |  | | | 63 | |  | | | | | | | 63 | |
| R^2^ Tjur | | |  | | | 0.019 | |  | | | | | | | 0.008 | | | | |  | | | | 0.093 | | | | |  | | | 0.008 | |  | | | | | | | 0.016 | |
|  |  |  | |  | | |  |  | | | | |  |  | |  | | | |  |  | |  | |  | | |  | |  |  | |  |  | | | | | |  |  |  |
|  |  |  | |  | | |  |  | | | | |  |  | |  | | | |  |  | |  | |  | | |  | |  |  | |  |  | | | | | |  |  |  |
| ***d) Teucrium* sp. nestling** | | | | | | | | | | | | | | | | | | | | | | | | | | | | | | | | | | | | | | | | | | |
|  | ***Plasmodium*** | | | | | | | | | ***Haemoproteus*** | | | | | | | ***Leucocytozoon*** | | | | | | | | ***Protocalliphora*** | | | | | | | | | | | | | ***Dermanyssus*** | | | | |
| *Predictors* | *Chis SQ* | *Log-Odds* | | *std. Error* | | | *p* | *Chis SQ* | | | | | *Log-Odds* | *std. Error* | | *p* | | | | *Chis SQ* | *Log-Odds* | | *std. Error* | | *p* | | | *Chis SQ* | | *Log-Odds* | *std. Error* | | *p* | *Chis SQ* | | | | | | *Log-Odds* | *std. Error* | *p* |
| (Intercept) |  | -4.90 | | 5.80 | | | 0.40 |  | | | | | -0.81 | 5.77 | | 0.89 | | | |  | 2.91 | | 6.71 | | 0.66 | | |  | | -2.47 | 6.00 | | 0.68 |  | | | | | | -4.61 | 6.58 | 0.48 |
| *Teucrium* sp. presence | 0.17 | -0.35 | | 0.87 | | | 0.69 | 0.38 | | | | | -0.54 | 0.87 | | 0.54 | | | | 0.69 | -0.45 | | 1.15 | | 0.70 | | | 0.00 | | 0.40 | 0.91 | | 0.66 | 0.11 | | | | | | 0.31 | 0.92 | 0.73 |
| Female mass | 1.03 | 0.55 | | 0.55 | | | 0.32 | 0.13 | | | | | 0.19 | 0.55 | | 0.73 | | | | 0.77 | -0.19 | | 0.63 | | 0.77 | | | 0.32 | | 0.31 | 0.58 | | 0.60 | 0.23 | | | | | | 0.30 | 0.63 | 0.63 |
| Laying date | 0.09 | -0.02 | | 0.08 | | | 0.76 | 0.35 | | | | | -0.05 | 0.08 | | 0.56 | | | | 0.08 | -0.17 | | 0.10 | | 0.10 | | | 0.10 | | -0.03 | 0.08 | | 0.75 | 0.23 | | | | | | 0.04 | 0.09 | 0.63 |
| Observations | | |  | | | 64 | |  | | | | | | | 64 | | | | |  | | | | 64 | | |  | | | | | 63 | |  | | | | | | | 63 | |
| R^2^ Tjur | | |  | | | 0.02 | |  | | | | | | | 0.014 | | | | |  | | | | 0.053 | | |  | | | | | 0.01 | |  | | | | | | | 0.009 | |
|  |  |  | |  | | |  |  | | | | |  |  | |  | | | |  |  | |  | |  | | |  | |  |  | |  |  | | | | | |  |  |  |
|  |  |  | |  | | |  |  | | | | |  |  | |  | | | |  |  | |  | |  | | |  | |  |  | |  |  | | | | | |  |  |  |
|  |  |  | |  | | |  |  | | | | |  |  | |  | | | |  |  | |  | |  | | |  | |  |  | |  |  | | | | | |  |  |  |
|  |  |  | |  | | |  |  | | | | |  |  | |  | | | |  |  | |  | |  | | |  | |  |  | |  |  | | | | | |  |  |  |
| ***e) Thymus* sp. nestling** | | | | | | | | | | | | | | | | | | | | | | | | | | | | | | | | | | | | | | | | | | |
|  | ***Plasmodium*** | | | | | | | | | | ***Haemoproteus*** | | | | | | | ***Leucocytozoon*** | | | | | | | | ***Protocalliphora*** | | | | | | | | | | | ***Dermanyssus*** | | | | | |
| *Predictors* | *Chis SQ* | *Log-Odds* | | *std. Error* | | | *p* | *Chis SQ* | | | | | *Log-Odds* | *std. Error* | | *p* | | | | *Chis SQ* | *Log-Odds* | | *std. Error* | | *p* | | | *Chis SQ* | | *Log-Odds* | *std. Error* | | *p* | *Chis SQ* | | | | | | *Log-Odds* | *std. Error* | *p* |
| (Intercept) |  | -5.11 | | 5.90 | | | 0.39 |  | | | | | -1.45 | 6.04 | | 0.81 | | | |  | 2.37 | | 6.50 | | 0.72 | | |  | | -3.05 | 6.08 | | 0.62 |  | | | | | | -4.13 | 6.90 | 0.55 |
| *Thymus* sp. presence | 0.27 | -0.28 | | 0.54 | | | 0.61 | 2.33 | | | | | -0.84 | 0.55 | | 0.13 | | | | 1.17 | -0.76 | | 0.73 | | 0.30 | | | 0.37 | | -0.33 | 0.55 | | 0.55 | 1.74 | | | | | | 0.78 | 0.59 | 0.19 |
| Female mass | 1.08 | 0.57 | | 0.56 | | | 0.31 | 0.22 | | | | | 0.27 | 0.57 | | 0.64 | | | | 0.05 | -0.13 | | 0.61 | | 0.83 | | | 0.42 | | 0.37 | 0.58 | | 0.52 | 0.13 | | | | | | 0.23 | 0.66 | 0.72 |
| Laying date | 0.06 | -0.02 | | 0.08 | | | 0.80 | 0.21 | | | | | -0.04 | 0.08 | | 0.64 | | | | 2.69 | -0.16 | | 0.10 | | 0.12 | | | 0.08 | | -0.02 | 0.08 | | 0.77 | 0.16 | | | | | | 0.04 | 0.09 | 0.69 |
| Observations | | |  | | | 64 | |  | | | | | | | 64 | | | | |  | | | | 64 | | | | |  | | | 63 | |  | | | | | | | 63 | |
| R^2^ Tjur | | |  | | | 0.021 | |  | | | | | | | 0.044 | | | | |  | | | | 0.065 | | | | |  | | | 0.013 | |  | | | | | | | 0.036 | |
|  |  |  | |  | | |  |  | | | | |  |  | |  | | | |  |  | |  | |  | | |  | |  |  | |  |  | | | | | |  |  |  |
| 1. ***Lamium* sp. before incubation** | | | | | | | | | | | | | | | | | | | | | | | | | | | | | | | | | | | | | | | | | | |
|  | ***Plasmodium*** | | | | | | | | | | ***Haemoproteus*** | | | | | | | ***Leucocytozoon*** | | | | | | | | ***Protocalliphora*** | | | | | | | | | | ***Dermanyssus*** | | | | | | |
| *Predictors* | *Chis SQ* | *Log-Odds* | | *std. Error* | | | *p* | *Chis SQ* | | | | | *Log-Odds* | *std. Error* | | *p* | | | | *Chis SQ* | *Log-Odds* | | *std. Error* | | *p* | | | *Chis SQ* | | *Log-Odds* | *std. Error* | | *p* | *Chis SQ* | | | | | | *Log-Odds* | *std. Error* | *p* |
| (Intercept) |  | -4.73 | | 5.81 | | | 0.42 |  | | | | | -0.79 | 5.84 | | 0.89 | | | |  | 2.03 | | 6.80 | | 0.77 | | |  | | -1.81 | 6.12 | | 0.77 |  | | | | | | -5.50 | 6.65 | 0.41 |
| *Lamium* sp. presence | 0.22 | -0.34 | | 0.72 | | | 0.64 | 0.23 | | | | | 0.36 | 0.77 | | 0.64 | | | | 2.76 | 1.26 | | 0.75 | | 0.09 | | | 4.36 | | -1.57 | 0.80 | | **0.048** | 1.31 | | | | | | 0.89 | 0.76 | 0.25 |
| Female mass | 1.01 | 0.54 | | 0.55 | | | 0.32 | 0.10 | | | | | 0.17 | 0.55 | | 0.76 | | | | 0.08 | -0.18 | | 0.63 | | 0.78 | | | 0.33 | | 0.33 | 0.58 | | 0.57 | 0.29 | | | | | | 0.34 | 0.63 | 0.59 |
| Laying date | 0.16 | -0.03 | | 0.08 | | | 0.69 | 0.20 | | | | | -0.04 | 0.08 | | 0.65 | | | | 1.66 | -0.14 | | 0.11 | | 0.21 | | | 0.79 | | -0.08 | 0.09 | | 0.38 | 0.59 | | | | | | 0.07 | 0.10 | 0.44 |
| Observations | | |  | | | 65 | |  | | | | | | | 65 | | | | |  | | | | 65 | | |  | | | | | 63 | |  | | | | | | | 63 | |
| R^2^ Tjur | | |  | | | 0.022 | |  | | | | | | | 0.011 | | | | |  | | | | 0.101 | | |  | | | | | 0.075 | |  | | | | | | | 0.03 | |
|  |  |  | |  | | |  |  | | | | |  |  | |  | | | |  |  | |  | |  | | |  | |  |  | |  |  | | | | | |  |  |  |
|  |  |  | |  | | |  |  | | | | |  |  | |  | | | |  |  | |  | |  | | |  | |  |  | |  |  | | | | | |  |  |  |
| ***f) Lamium* sp. incubation** | | | | | | | | | | | | | | | | | | | | | | | | | | | | | | | | | | | | | | | | | | |
|  | ***Plasmodium*** | | | | | | | | | | ***Haemoproteus*** | | | | | | | ***Leucocytozoon*** | | | | | | | | ***Protocalliphora*** | | | | | | | | | ***Dermanyssus*** | | | | | | | |
| *Predictors* | *Chis SQ* | *Log-Odds* | | *std. Error* | | | *p* | *Chis SQ* | | | | | *Log-Odds* | *std. Error* | | *p* | | | | *Chis SQ* | *Log-Odds* | | *std. Error* | | *p* | | | *Chis SQ* | | *Log-Odds* | *std. Error* | | *p* | *Chis SQ* | | | | | | *Log-Odds* | *std. Error* | *p* |
| (Intercept) |  | -5.06 | | 5.81 | | | 0.38 |  | | | | | -0.61 | 5.79 | | 0.92 | | | |  | 2.53 | | 6.80 | | 0.71 | | |  | | -2.26 | 6.04 | | 0.71 |  | | | | | | -4.15 | 6.61 | 0.53 |
| *Lamium* sp. presence | 0.01 | -0.07 | | 0.83 | | | 0.93 | 0.15 | | | | | -0.33 | 0.83 | | 0.70 | | | | 0.70 | -0.90 | | 1.16 | | 0.44 | | | 0.49 | | 0.61 | 0.90 | | 0.50 | 0.97 | | | | | | 0.86 | 0.85 | 0.32 |
| Female mass | 1.06 | 0.56 | | 0.55 | | | 0.31 | 0.11 | | | | | 0.18 | 0.55 | | 0.75 | | | | 0.04 | -0.13 | | 0.64 | | 0.84 | | | 0.22 | | 0.27 | 0.58 | | 0.64 | 0.13 | | | | | | 0.23 | 0.63 | 0.72 |
| Laying date | 0.09 | -0.02 | | 0.08 | | | 0.77 | 0.40 | | | | | -0.05 | 0.08 | | 0.53 | | | | 3.42 | -0.19 | | 0.11 | | 0.08 | | | 0.05 | | -0.02 | 0.08 | | 0.82 | 0.38 | | | | | | 0.06 | 0.09 | 0.54 |
| Observations | | |  | | | 65 | |  | | | | | | | 65 | | | | |  | | | | 65 | | | | |  | | | 63 | |  | | | | | | | 63 | |
| R^2^ Tjur | | |  | | | 0.019 | |  | | | | | | | 0.01 | | | | |  | | | | 0.058 | | | | |  | | | 0.015 | |  | | | | | | | 0.024 | |
|  |  |  | |  | | |  |  | | | | |  |  | |  | | | |  |  | |  | |  | | |  | |  |  | |  |  | | | | | |  |  |  |
|  |  |  | |  | | |  |  | | | | |  |  | |  | | | |  |  | |  | |  | | |  | |  |  | |  |  | | | | | |  |  |  |
| ***f) Lamium* sp. nestling** | | | | | | | | | | | | | | | | | | | | | | | | | | | | | | | | | | | | | | | | | | |
|  | ***Plasmodium*** | | | | | | | | | | | ***Haemoproteus*** | | | | | | | ***Leucocytozoon*** | | | | | | | ***Protocalliphora*** | | | | | | | | | ***Dermanyssus*** | | | | | | | |
| *Predictors* | *Chis SQ* | *Log-Odds* | | *std. Error* | | | *p* | *Chis SQ* | | | | | *Log-Odds* | *std. Error* | | *p* | | | | *Chis SQ* | *Log-Odds* | | *std. Error* | | *p* | | | *Chis SQ* | | *Log-Odds* | *std. Error* | | *p* | *Chis SQ* | | | | | | *Log-Odds* | *std. Error* | *p* |
| (Intercept) |  | -6.64 | | 6.23 | | | 0.29 |  | | | | | -1.85 | 6.07 | | 0.76 | | | |  | 2.03 | | 6.86 | | 0.77 | | |  | | -4.02 | 6.38 | | 0.53 |  | | | | | | -4.29 | 6.81 | 0.53 |
| *Lamium* sp. presence | 3.70 | 16.51 | | 1335.99 | | | 0.99 | 2.82 | | | | | 16.15 | 1377.13 | | 0.99 | | | | 1.42 | 1.52 | | 1.32 | | 0.25 | | | 3.44 | | 16.42 | 1365.41 | | 0.99 | 1.77 | | | | | | -15.57 | 1377.06 | 0.99 |
| Female mass | 1.34 | 0.66 | | 0.59 | | | 0.26 | 0.18 | | | | | 0.24 | 0.57 | | 0.67 | | | | 0.06 | -0.15 | | 0.64 | | 0.81 | | | 0.47 | | 0.41 | 0.61 | | 0.50 | 0.23 | | | | | | 0.31 | 0.65 | 0.63 |
| Laying date | 0.02 | 0.01 | | 0.08 | | | 0.89 | 0.04 | | | | | -0.02 | 0.08 | | 0.83 | | | | 1.83 | -0.14 | | 0.11 | | 0.19 | | | 0.01 | | 0.01 | 0.08 | | 0.93 | 0.04 | | | | | | 0.02 | 0.09 | 0.85 |
| Observations | | |  | | | 64 | |  | | | | | | | 64 | | | | |  | | | | 64 | | | | |  | | | 63 | |  | | | | | | | 63 | |
| R^2^ Tjur | | |  | | | 0.058 | |  | | | | | | | 0.036 | | | | |  | | | | 0.079 | | | | |  | | | 0.045 | |  | | | | | | | 0.023 | |
|  |  |  | |  | | |  |  | | | | |  |  | |  | | | |  |  | |  | |  | | |  | |  |  | |  |  | | | | | |  |  |  |
|  |  |  | |  | | |  |  | | | | |  |  | |  | | | |  |  | |  | |  | | |  | |  |  | |  |  | | | | | |  |  |  |

**Table S4.** Final Generalized linear models (GLMs) with negative binomial distribution showing the effects of the presence of green plants per separate species (i.e., a) *Achillea* sp., b) *Lavandula* sp., c) *Anthriscus* sp., d) *Teucrium* sp., e) *Thymus* sp., f) *Lamium* sp., g) *Clinopodium* sp.) on the number of blowflies *Protocalliphora* *azurea* provided by blue tit females before the incubation, incubation and nestling period. Significant effects (P < 0.05) are marked in bold.

|  | *Achillea* sp. | | | | | | | | | | | |
| --- | --- | --- | --- | --- | --- | --- | --- | --- | --- | --- | --- | --- |
|  | **before incubation** | | | | **incubation** | | | | **nestling** | | | |
| *Predictors* | *Chis SQ* | *Log-Odds* | *std. Error* | *p* | *Chis SQ* | *Log-Odds* | *std. Error* | *p* | *Chis SQ* | *Log-Odds* | *std. Error* | *p* |
| (Intercept) |  | 0.29 | 5.42 | 0.96 |  | -0.49 | 5.4 | 0.93 |  | 0.23 | 5.37 | 0.97 |
| *Achillea* sp. presence | 0.99 | -0.01 | 0.66 | 0.99 | 0.16 | -0.25 | 0.62 | 0.68 | 0.99 | -0.82 | 0.76 | 0.28 |
| Female mass | 0.85 | 0.09 | 0.52 | 0.87 | 0.09 | 0.18 | 0.52 | 0.74 | 0.03 | 0.11 | 0.51 | 0.83 |
| Laying date | 0.58 | 0.04 | 0.07 | 0.58 | 0.22 | 0.04 | 0.07 | 0.64 | 0.17 | 0.03 | 0.07 | 0.68 |
| Observations | 62 | | | | 63 | | | | 63 | | | |
| R^2^ Tjur | 0.045 | | | | 0.01 | | | | 0.008 | | | |
|  | *Lavandula* sp. | | | | | | | | | | | |
|  | **before incubation** | | | | **incubation** | | | | **nestling** | | | |
| *Predictors* | *Chis SQ* | *Log-Odds* | *std. Error* | *p* | *Chis SQ* | *Log-Odds* | *std. Error* | *p* | *Chis SQ* | *Log-Odds* | *std. Error* | *p* |
| (Intercept) |  | -0.18 | 5.4 | 0.97 |  | 1.09 | 5.53 | 0.84 |  | 1.58 | 5.38 | 0.77 |
| *Lavandula* sp. presence | 0.81 | 0.14 | 0.59 | 0.81 | 2.76 | 0.82 | 0.53 | 0.13 | 1.43 | 0.62 | 0.48 | 0.2 |
| Female mass | 0.82 | 0.14 | 0.52 | 0.79 | 0.004 | -0.04 | 0.54 | 0.95 | 0.01 | -0.07 | 0.52 | 0.9 |
| Laying date | 0.64 | 0.03 | 0.08 | 0.65 | 0.51 | 0.05 | 0.07 | 0.5 | 0.15 | 0.03 | 0.07 | 0.72 |
| Observations | 63 | | | | 63 | | | | 63 | | | |
| R^2^ Tjur | 0.009 | | | | 0.017 | | | | 0.009 | | | |
|  |  | | | | | | | | | | | |
|  |  | | | | | | | | | | | |
|  |  | | | | | | | | | | | |
|  | *Anthriscus* sp. | | | | | | | | | | | |
|  | **before incubation** | | | | **incubation** | | | | **nestling** | | | |
| *Predictors* | *Chis SQ* | *Log-Odds* | *std. Error* | *p* | *Chis SQ* | *Log-Odds* | *std. Error* | *p* | *Chis SQ* | *Log-Odds* | *std. Error* | *p* |
| (Intercept) |  | 0.6 | 5.39 | 0.91 |  | -0.7 | 5.47 | 0.9 |  | 0.72 | 5.41 | 0.89 |
| *Anthriscus* sp. presence | 0.18 | 0.23 | 0.52 | 0.66 | 0.02 | -0.1 | 0.68 | 0.88 | 0.3 | 0.29 | 0.51 | 0.57 |
| Female mass | 0.01 | 0.06 | 0.52 | 0.91 | 0.09 | 0.19 | 0.52 | 0.71 | 0.01 | 0.05 | 0.52 | 0.92 |
| Laying date | 0.17 | 0.03 | 0.08 | 0.69 | 0.24 | 0.04 | 0.08 | 0.64 | 0.12 | 0.03 | 0.07 | 0.73 |
| Observations | 63 | | | | 63 | | | | 63 | | | |
| R^2^ Tjur | 0.012 | | | | 0.02 | | | | 0.013 | | | |
|  | *Teucrium* sp. | | | | | | | | | | | |
|  | **before incubation** | | | | **incubation** | | | | **nestling** | | | |
| *Predictors* | *Chis SQ* | *Log-Odds* | *std. Error* | *p* | *Chis SQ* | *Log-Odds* | *std. Error* | *p* | *Chis SQ* | *Log-Odds* | *std. Error* | *p* |
| (Intercept) |  | -0.42 | 5.28 | 0.94 |  | -0.51 | 5.4 | 0.93 |  | -0.16 | 5.41 | 0.98 |
| *Teucrium* sp. presence | 2.2 | -1.29 | 0.77 | 0.094 | 0.04 | -0.17 | 0.88 | 0.85 | 0.04 | 0.16 | 0.8 | 0.84 |
| Female mass | 0.07 | 0.16 | 0.51 | 0.76 | 0.08 | 0.18 | 0.52 | 0.74 | 0.05 | 0.14 | 0.52 | 0.79 |
| Laying date | 0.43 | 0.05 | 0.07 | 0.51 | 0.21 | 0.03 | 0.08 | 0.65 | 0.24 | 0.04 | 0.07 | 0.63 |
| Observations | 63 | | | | 63 | | | | 63 | | | |
| R^2^ Tjur | 0.051 | | | | 0.008 | | | | 0.01 | | | |
|  |  |  |  |  |  |  |  |  |  |  |  |  |
|  | *Lamium* sp. | | | | | | | | | | | |
|  | **before incubation** | | | | **incubation** | | | | **nestling** | | | |
| *Predictors* | *Chis SQ* | *Log-Odds* | *std. Error* | *p* | *Chis SQ* | *Log-Odds* | *std. Error* | *p* | *Chis SQ* | *Log-Odds* | *std. Error* | *p* |
| (Intercept) |  | 0.27 | 5.31 | 0.96 |  | -0.4 | 5.43 | 0.94 |  | 0.31 | 5.42 | 0.96 |
| *Lamium* sp. presence | 2.6 | -1.18 | 0.67 | 0.079 | 0.03 | -0.14 | 0.76 | 0.85 | 0.69 | -1.1 | 1.19 | 0.36 |
| Female mass | 0.07 | 0.13 | 0.51 | 0.79 | 0.08 | 0.17 | 0.52 | 0.75 | 0.04 | 0.11 | 0.52 | 0.83 |
| Laying date | 0.04 | 0.02 | 0.08 | 0.85 | 0.19 | 0.03 | 0.08 | 0.66 | 0.1 | 0.02 | 0.08 | 0.76 |
| Observations | 63 | | | | 63 | | | | 63 | | | |
| R^2^ Tjur | 0.075 | | | | 0.015 | | | | 0.045 | | | |
|  |  | | | | | | | | | | | |
| *Thymus* sp. | | | |  |  |  |  |  |  |  |  |  |
| **nestling** | | | |  |  |  |  |  |  |  |  |  |
| *Chis SQ* | *Log-Odds* | *std. Error* | *p* |  |  |  |  |  |  |  |  |  |
|  | -1.16 | 5.41 | 0.83 |  |  |  |  |  |  |  |  |  |
| 0.16 | -0.21 | 0.5 | 0.67 |  |  |  |  |  |  |  |  |  |
| 0.14 | 0.24 | 0.52 | 0.64 |  |  |  |  |  |  |  |  |  |
| 0.24 | 0.04 | 0.07 | 0.63 |  |  |  |  |  |  |  |  |  |
| 63 | | | |  |  |  |  |  |  |  |  |  |
| 0.013 | | | |  |  |  |  |  |  |  |  |  |
|  |  |  |  |  |  |  |  |  |  |  |  |  |
